# Supplementary material for: A deep learning model for predicting next-generation sequencing depth from DNA sequence
Source: Nat Commun. 2021 Jul 19;12:4387. doi: 10.1038/s41467-021-24497-8 (PMC8290051; doi:10.1038/s41467-021-24497-8)
Supplement: Supplementary file 1 — Supplementary Information [file 41467_2021_24497_MOESM1_ESM.pdf]

A Deep Learning Model for Predicting Next-Generation Sequencing Depth from DNA Sequence

Jinny X. Zhang*,[1,2] Boyan Yordanov*,[3,4] Alexander Gaunt*,[3] Michael X. Wang*,[1] Peng Dai,[1] Yuan-Jyue Chen,[5] Kerou Zhang,[1] John Z. Fang,[1] Neil Dalchau,[3] Jiaming Li,[1,2] Andrew Phillips+,[3] and David Yu Zhang+[1,2]

[1]*Department of Bioengineering, Rice University, Houston, TX*
[2]*Systems, Synthetic, and Physical Biology, Rice University, Houston, TX*
[3]*Microsoft Research, Cambridge, UK*
[4]*Scientific Technologies, London UK*
[5]*Microsoft Research, Seattle, WA*
* *these authors contributed equally to this work*
+ *correspondence and requests for materials should be addressed to AP (email: Andrew.Phillips@microsoft.com) and DYZ (email: dyz1@rice.edu)*

(Dated: June 3, 2021)

Supplementary Note

1. DLM Mathematical Details 3
2. Strand Displacement Kinetics Experiment and Rate Constant Fitting 5
3. Additional Experiments 32
4. Contributions to the DLM Performance 41
5. Random Guess Models of Kinetics 43

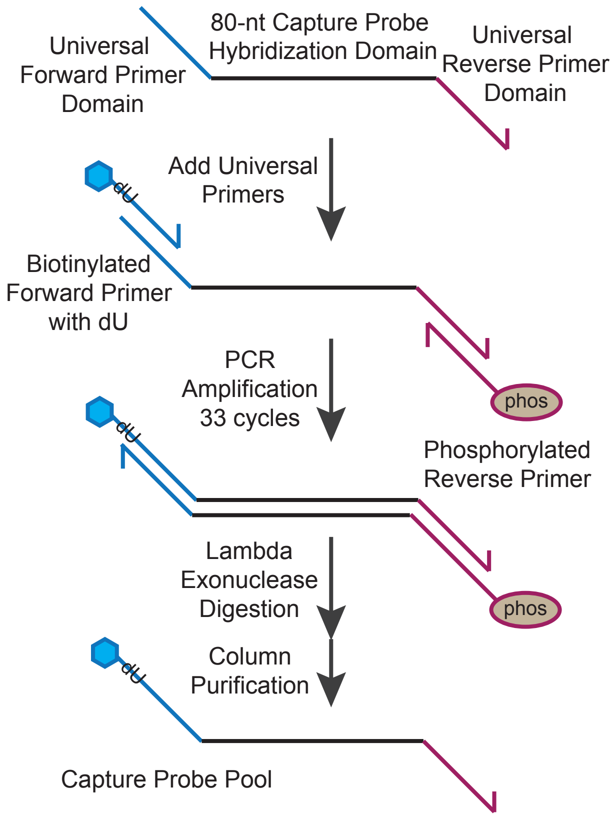

FIG. S1: Capture probe preparation protocol.

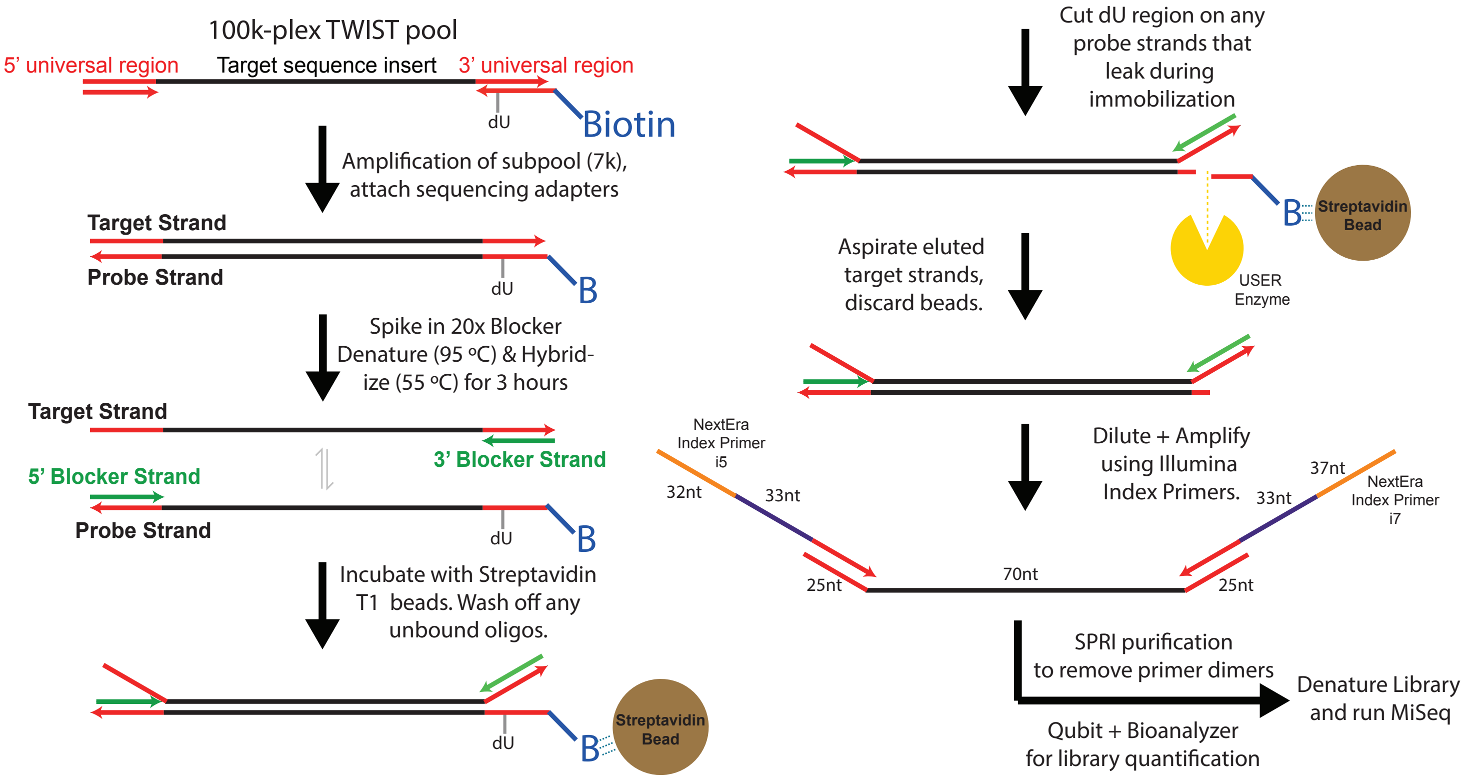

FIG. S2: Synthetic DNA NGS experiment workflow.

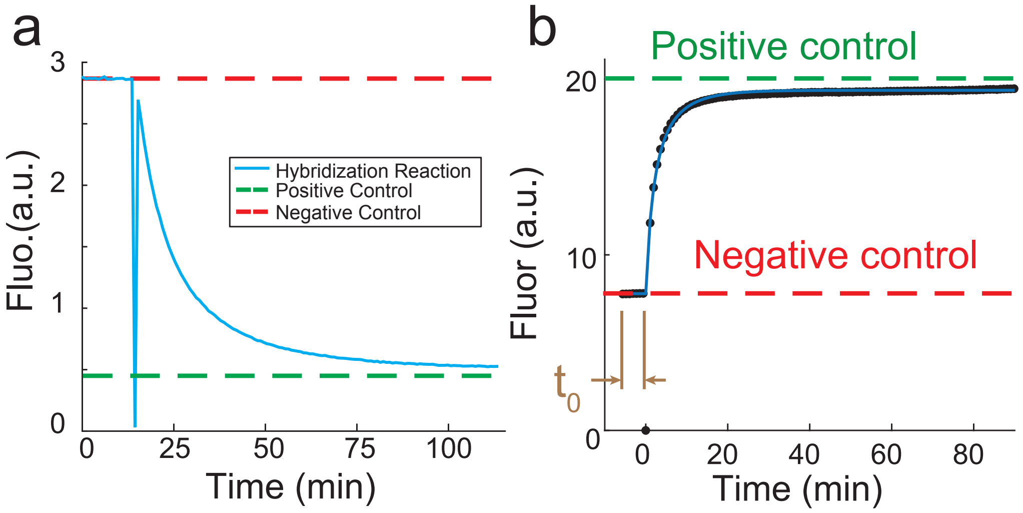

FIG. S3: Sample kinetics reaction traces and measured positive(green dash) and negative controls(red dash). **(a)** Hybridization trace. **(b)** Strand displacement trace.

Supplementary Note 1. DLM Mathematical Details

Mathematically, the local features of a strand are represented by a three element vector $\mathbf{x}_t$ for each base position t of the strand of length L, where $1 \leq t \leq L$. The first element of each vector is the probability p_{open} that the base is not paired prior to target-probe mixing, and the second and third element are the binary base identity with 00 represents T, 01 represents C, 10 represent A and 11 represents G. P_{open} considers arbitrary secondary structures and encodes the expected availability of each base to interact, whereas binary base identity as a low level feature allows the DLM to dig other information that is not provided by open probability. Features $\mathbf{x}_t$ are computed for all bases on both the target and probe strands using Nupack.

The global features supplied to the DLM represent reaction conditions and energy scales, and comprise a four element vector $\mathbf{X}_{\text{glob}}$. This vector includes the reaction temperature T and the free energies of the target $\Delta G^\circ(T)$, probe $\Delta G^\circ(P)$, and resulting target-probe complex $\Delta G^\circ(TP)$. The values of free energy features ΔG are calculated for DNA strands and complexes following ref. [1, 2], using the Nupack software [3]. The standard free energies features are included because they offer a tractable proxy for the rough scale of the reaction activation energy that cannot be easily estimated from the local features described above. Arrhenius arguments suggest that this energy scale should be important in estimating reaction rates, and in Supplementary Section S4 we indeed find that $\Delta G^\circ(P)$ in particular has an effect on our model predictions of hybridization and strand displacement rate constants. This is consistent with reaction mechanisms in which the disruption of the target strand's equilibrium secondary structure is a significant fraction of the overall reaction time.

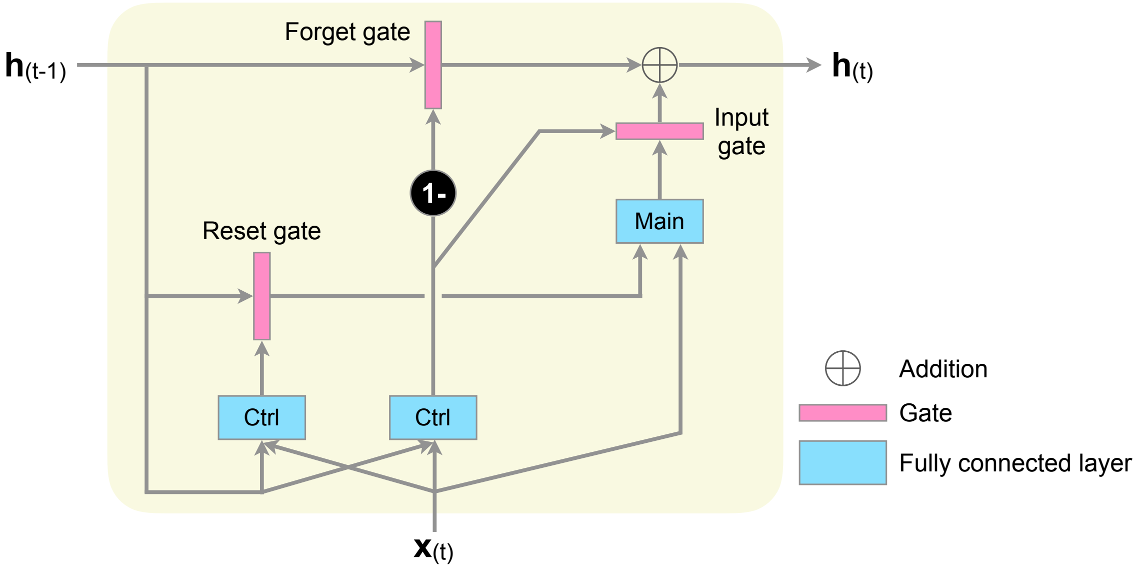

FIG. S4: The hidden vector $\mathbf{h}$ serves as the "memory" of a GRU that is updated with each input vector $\mathbf{x}$ based on learnable weights $\mathbf{W}$ in the fully connected layers. At each step t, part of $\mathbf{h}_{t-1}$ is erased and then filled with new memories, determined by two "gates": forget gate and input gate which are controlled by the one same fully connected layer taking $\mathbf{h}_{t-1}$ and $\mathbf{x}_t$ as input. The content of the new memoties is calculated based on $\mathbf{x}_t$ and $\mathbf{h}_{t-1}$ by the main layer, another fully connected layer inside GRU. However, only part of $\mathbf{h}_{t-1}$ is presented to the main layer, which is determined by the reset gate that is also controlled by a fully connected layer. A GRU could switfly adapt to new input by changing part of the vector $\mathbf{h}$ while keeping some "long-term" features at the same time.

The RNN is implemented using a Gated Recurrent Unit (GRU) [4], parametrized by learnable weights $\mathbf{W}$, that is run over the local features $\mathbf{x}_t$. A simple illustration of the The recurrent action of this RNN produces a sequence of latent representations $\mathbf{h}_t$

$$\mathbf{h}_t = GRU(\mathbf{h}_{t-1}, \mathbf{x}_t; \mathbf{W}), \tag{2}$$

where we initialize $\mathbf{h}_0 = \mathbf{0}$. Fig. S1 shows a simple illustration of the GRU we used in our DLM. We keep only the final vector $\mathbf{H} = \mathbf{h}_L$ produced at $t = L$ as a summary of the entire strand.

The RNN runs separately over the target and probe strands in both $5'$ to $3'$ and $3'$ to $5'$ direction to produce latent vectors summarizing each strand $\mathbf{H}_T^{5'->3'}$, $\mathbf{H}_P^{5'->3'}$, $\mathbf{H}_T^{3'->5'}$ and $\mathbf{H}_P^{3'->5'}$. We concatenate $\mathbf{H}_T^{5'->3'} + \mathbf{H}_P^{5'->3'}$ and $\mathbf{H}_T^{3'->5'} + \mathbf{H}_P^{3'->5'}$, then concatenate with the 4-element global feature vector, $\mathbf{X}_{\text{glob}}$, to obtain an input

$[\mathbf{H}_T^{5'->3'} + \mathbf{H}_P^{5'->3'}, \mathbf{H}_T^{3'->5'} + \mathbf{H}_P^{3'->5'}, \mathbf{X}_{\text{glob}}]$. Since hybridization can begin at either end or in the middle of the target strand, considering both directions easures that our model does not overconsider the bases near $5'$ end or $3'$ end. In preliminary experiments based on 8-fold cross validation, we obtained good results by choosing $\mathbf{h}_t$ to be 128-dimensional and the hidden dimensions of the output network layers to be 256 and 128.

Supplementary Note 2. Strand Displacement Kinetics Experiment and Rate Constant Fitting

DNA strand displacement is a competitive reaction in which a single-stranded target oligonucleotide T binds to a partially double-stranded probe complex comprising a complementary oligonucleotide C bound to a shorter protector oligonucleotide P. The nucleotides in C that are complementary to T but complementary to P are collectively known as the *toehold*, and serves to initiate the strand displacement reaction [5].

Here, we measure the kinetics of 211 strand displacement reactions across 100 target sequences (8 experiments at 28 °C, 99 experiments at 37 °C, 8 experiments at 46 °C, 96 experiments at 55 °C). The target sequences are 36 nt subsequences of the human *CYCS* and *VEGF* genes. Furthermore, these are the same target sequences as the hybridization experiments used in ref. [6], to facilitate a direct comparison of hybridization and strand displacement kinetics.

We measured strand displacement kinetics by observing fluorescence time courses using the X-probe architecture (manuscript Fig. 2A), in order to minimize the number of unique chemically modified oligonucleotides, following principles described in ref. [7]. The probe initially exhibits low fluorescence due to the co-localization of the fluorophore and quencher, but increases in fluorescence after hybridization to the target. Given the concentrations of the oligonucleotide species involved, we can fit the rate constant of strand displacement (k_{dsp}). The length of the toehold ($\mathrm{b_{toe}}$) for each strand displacement reaction was selected such that the standard free energy of toehold binding ($\Delta G^{\circ}_{\mathrm{toe}}$) was approximately -10 kcal/mol (Fig. S5b), following previous studies showing that strand displacement kinetics saturate at this strength [5]. Because the standard free energy of DNA base pairing is weaker at higher temperatures, longer length toeholds were used for strand displacement reactions at 55 °C compared to 37 °C.

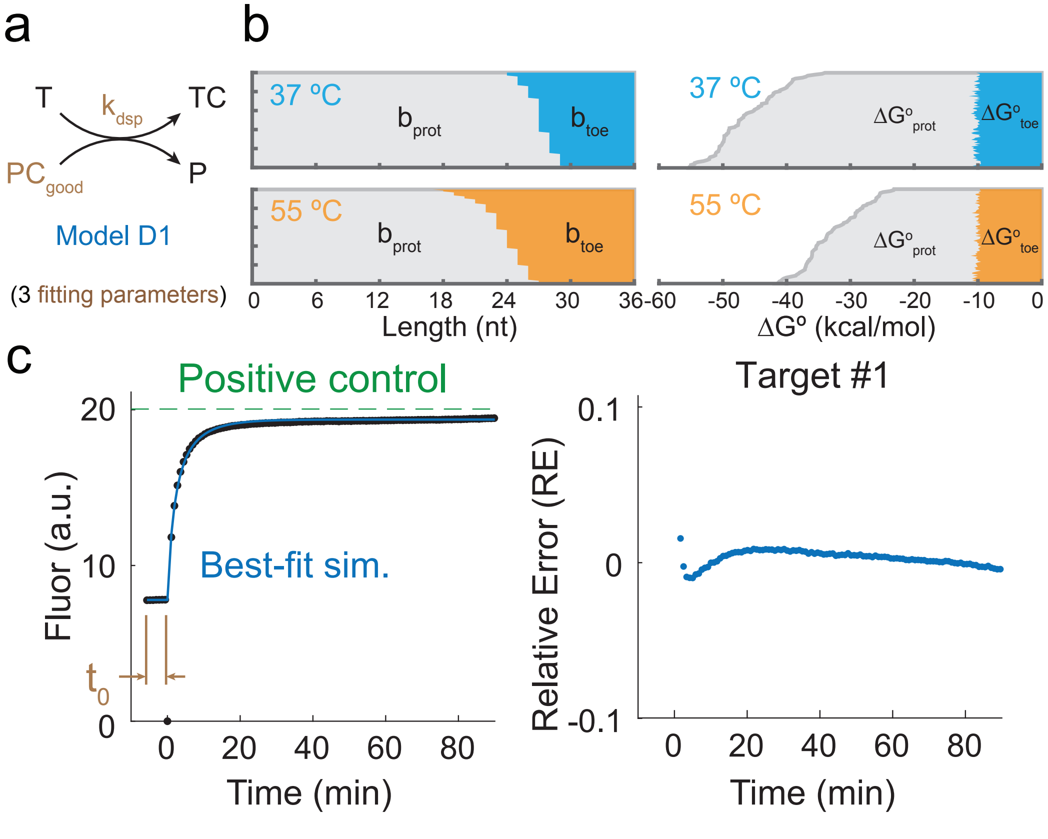

FIG. S5: DNA strand displacement reaction model and fitting. **(a)** Reaction model for fitting strand displacement rate constants. Three parameters are fitted to each kinetics trace: the rate constant k_{dsp}, the fraction of probe PC that is well-synthesized and capable of strand displacement, and the time (t_0) of addition of the trigger strand to the reaction volume. **(b)** Distribution of base pair length of protector and toehold(left panel) and computed $\Delta G^{\circ}_{\mathrm{prot}}$ and $\Delta G^{\circ}_{\mathrm{toe}}$ values(right panel) for each strand displacement reaction. **(c)** Relative error of fitting at each time point.

Strand displacement kinetics and rate constant fitting.

Inferring k_{dsp} from kinetics relies on an assumed reaction model. Briefly, we perform ordinary differential

equation simulations of the reaction model for particular values of fitting parameters including k_{dsp}, and calculate the root mean square relative error (RMSRE), where the relative error is defined as $\frac{F_{\mathrm{obs}}-F_{\mathrm{sim}}}{F_{\mathrm{obs}}}$, where F_{obs} is the observed fluorescence and F_{sim} is the simulated fluorescence. Different reaction models can be compared against each other via the RMSRE using best-fit parameters, and the simplest reaction model that gives the lowest RMSRE is considered to be the best fit to the data.

Evaluation of several different models against the experimental data (next subsection) shows that model D1 (Fig. S1a) provides the best fit with the smallest number of fitting parameters. The D1 model assumes three fitted parameters for each reaction: k_{dsp}, the concentration of well-synthesized probe molecules $[\mathrm{PC}_{\mathrm{good}}]$, and the time t_0 at which the reaction was began through injection of the target solution. The parameter $[\mathrm{PC}_{\mathrm{good}}]$ is intended to capture the effects of imperfectly synthesized oligonucleotides, that are not capable of rapid strand displacement. For example, chemical synthesis of DNA oligonucleotides often results in truncations at the $5'$ end, which erode the toehold and render the strand displacement reaction both kinetically slower and thermodynamically less favorable. The fitting of t_0 captures the fact that the manually recorded start time of each reaction does not exactly correspond to the actual reaction start time, due to inevitable delays in experimental steps. Fitting rate constants based on the nominal start time (as in ref. [6]) may result in significant errors when the reaction rate constant is very fast (i.e. the reaction is nearly complete by the time of the first fluorescence measurement).

Reaction Model Fitting

Models. We considered three strand displacement reaction models D1, D1m, D2 and D2m, with the following modeled reactions:

Model D1. A one-step strand displacement reaction with kinetic parameter k_{dsp}, and a second parameter f_{good} describing the fraction of correctly assembled probes.

$$\begin{aligned} T + C_{\mathrm{good}} &\overset{k_{\mathrm{dsp}}}{\rightarrow} D + P \\ \frac{[P]_0 - [P_{\mathrm{good}}]_0}{[P]_0} &= \text{Bad Fraction} \end{aligned}$$

Model D1m. A two-step strand displacement reaction with kinetic parameters k^1_{dsp}, k^2_{dsp}, and a third parameter f_{good} describing the fraction of correctly assembled probes.

$$\begin{aligned} T + C_{\mathrm{good}} &\overset{k^1_{\mathrm{dsp}}}{\rightarrow} TC \\ TC &\overset{k^2_{\mathrm{dsp}}}{\rightarrow} D + P \\ \frac{[P]_0 - [P_{\mathrm{good}}]_0}{[P]_0} &= \text{Bad Fraction} \end{aligned}$$

Model D2. A one-step strand displacement reaction with kinetic parameter k_{dsp}, and additional kinetic parameters k_1 and k_2 describing conformational changes of the target strand.

$$\begin{aligned} T &\underset{k_2}{\overset{k_1}{\rightleftharpoons}} T^* \\ T + C &\overset{k_{\mathrm{dsp}}}{\rightarrow} D + P \end{aligned}$$

Model D2m. A two-step strand displacement reaction with kinetic parameters k^1_{dsp}, k^2_{dsp}, and additional kinetic parameters k_1 and k_2 describing conformational changes of the target strand.

$$\begin{aligned} T &\underset{k_2}{\overset{k_1}{\rightleftharpoons}} T^* \\ T + C &\overset{k^1_{\mathrm{dsp}}}{\rightarrow} TC \\ TC &\overset{k^2_{\mathrm{dsp}}}{\rightarrow} D + P \end{aligned}$$

Performance comparison. For a fair comparison of the four models against one another, we obtain the best-fit parameter values of each model for each strand displacement experiment. We used the same parameterization

methodology as described in [6]. The performance of each model fit is quantified by the root mean square relative error (RMSRE) in Fig. S1. We also compared whether the time of treatment of the target strand (t_0) was fixed (at the time of the first measurement following treatment; Fig. S6a) or inferred (Fig. S6b). We found that inferring t_0 improved the RMSRE score for D1, D1m and D2m, but decreased performance for D2m. Nevertheless, D1 and D1m were the better models overall in terms of their RMSRE scores.

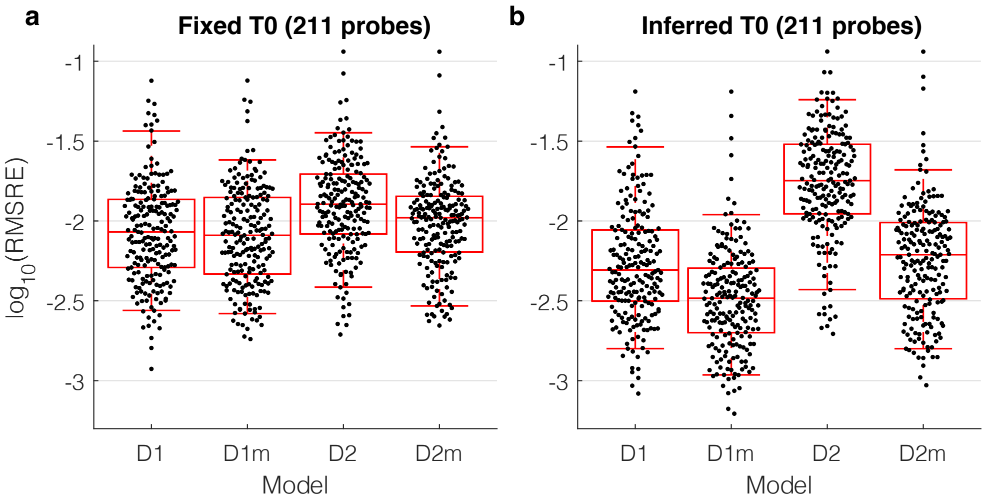

FIG. S6: **Comparison of reaction models performance, measured by root mean square relative error.** In box-whisker plots, the central mark indicates the median, and the bottom and top edges of the box indicate the 25th and 75th percentiles, respectively. The maximum whisker lengths are specified as 1.5 times the interquartile range.

Because each model and t_0 choice led to different numbers of parameters being inferred, we compared the Bayesian information criterion statistic to determine whether additional parameters were justified (Fig. S7). As D1 and D1m had fewer parameters than D2 and D2m respectively, the BIC scores continued to favor those models. However, we found that increased RMSRE performance of inferring t_0 was only observable in the BIC statistic for D1 (mean BIC scores of 3.12 v.s. 3.09) and D1m (mean BIC scores of 3.11 v.s. 3.06). Therefore, overall D1m had a marginally better BIC score than D1.

In this article, we construct a model that predicts the rate of strand displacement. The analysis of this section suggests that our measurements of strand displacement favor a two-step model scheme, but this would be harder to predict, as the contributions to strand displacement would be split over two quantities. Therefore, we decided to proceed using the parameters from the D1 hypothesis, which enables us to abstract the rate as a single quantity, with only a small loss of performance in describing our measurements. As such, we leave the prediction of strand displacement reactions as a two-step process as future work.

Time-series comparison. The raw fluorescence data and the best-fit traces for each model are shown in Fig. S8 through S19. Because the simulation traces for the three models appear very similar for many strand displacement experiments, Fig. S20 through S31 show the relative error RE $= \text{Abs}\left(\frac{s_i - y_i}{y_i}\right)$ of each model for each strand displacement experiment. Note that some figure subpanels are empty (e.g. Target #30 at 55 °C in Fig. S16) because the strand displacement reaction was not thermodynamically favorable for the target and probe sequence pair at the listed temperature. For these experiments, no significant increase of fluorescence was observed upon addition of the T strand.

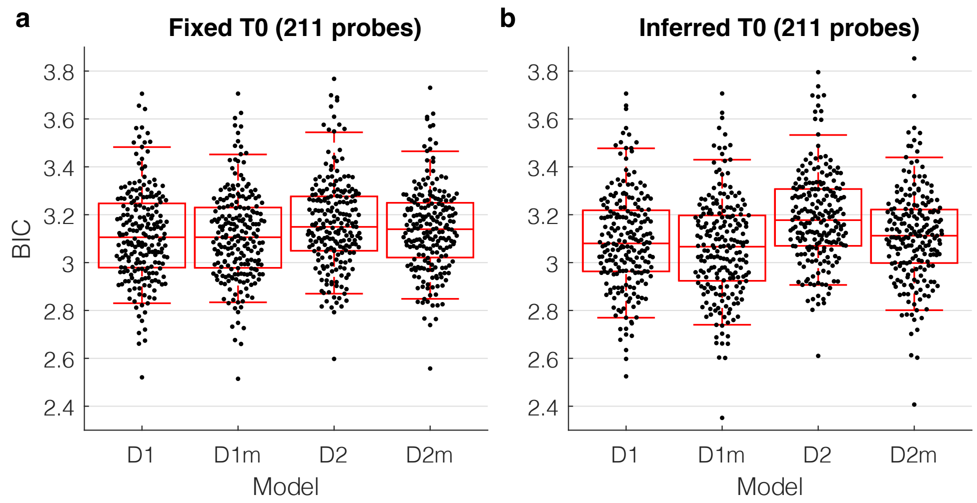

FIG. S7: **Comparison of reaction models performance, measured by the Bayesian information criterion (BIC).** The BIC is equal to $-2 \cdot l(\theta^*) + k \log d$ where $l(\theta^*)$ is the log-likelihood of the maximizing parameters θ^*, k is the number of parameters to be inferred and d is the number of data-points. In box-whisker plots, the central mark indicates the median, and the bottom and top edges of the box indicate the 25th and 75th percentiles, respectively. The maximum whisker lengths are specified as 1.5 times the interquartile range.

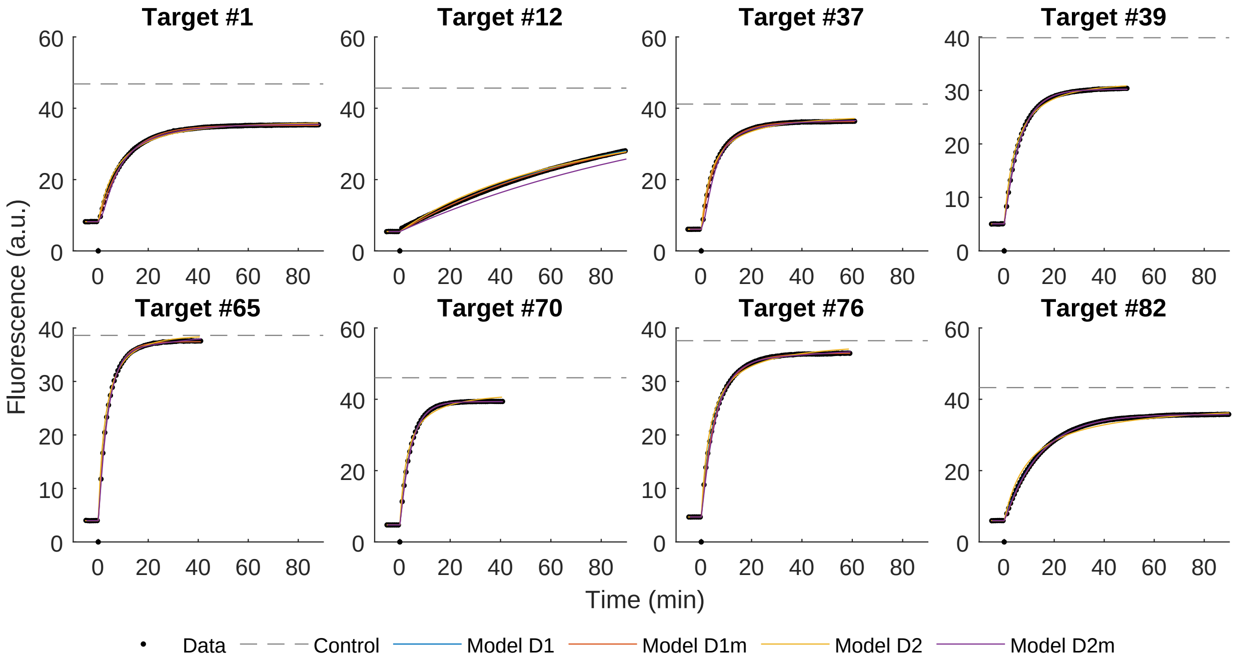

FIG. S8: **Fluorescence data and best-fit traces.** Strand displacement experiments performed at 28 °C.

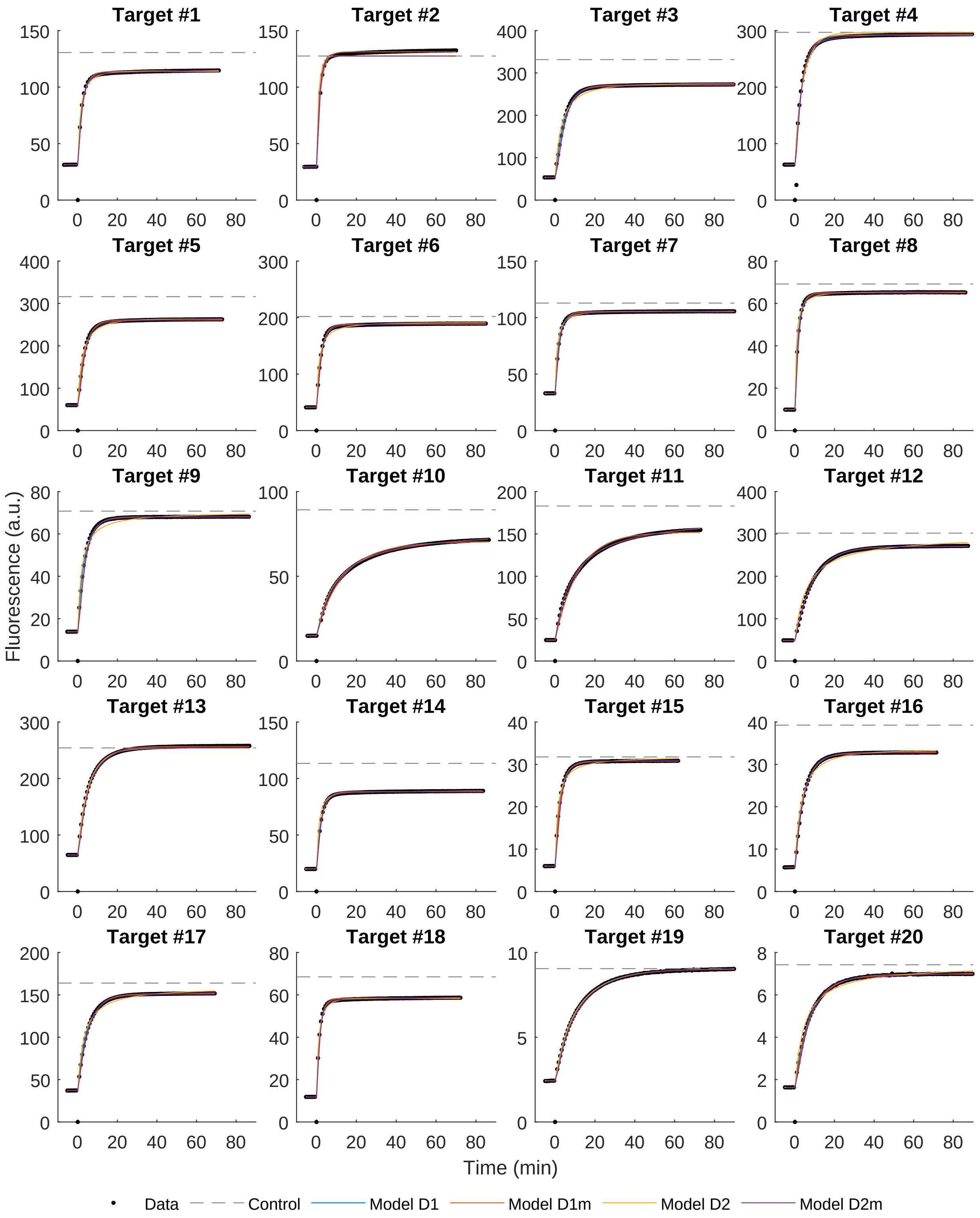

FIG. S9: **Fluorescence data and best-fit traces.** Strand displacement experiments performed at 37 °C, target sequences 1-20.

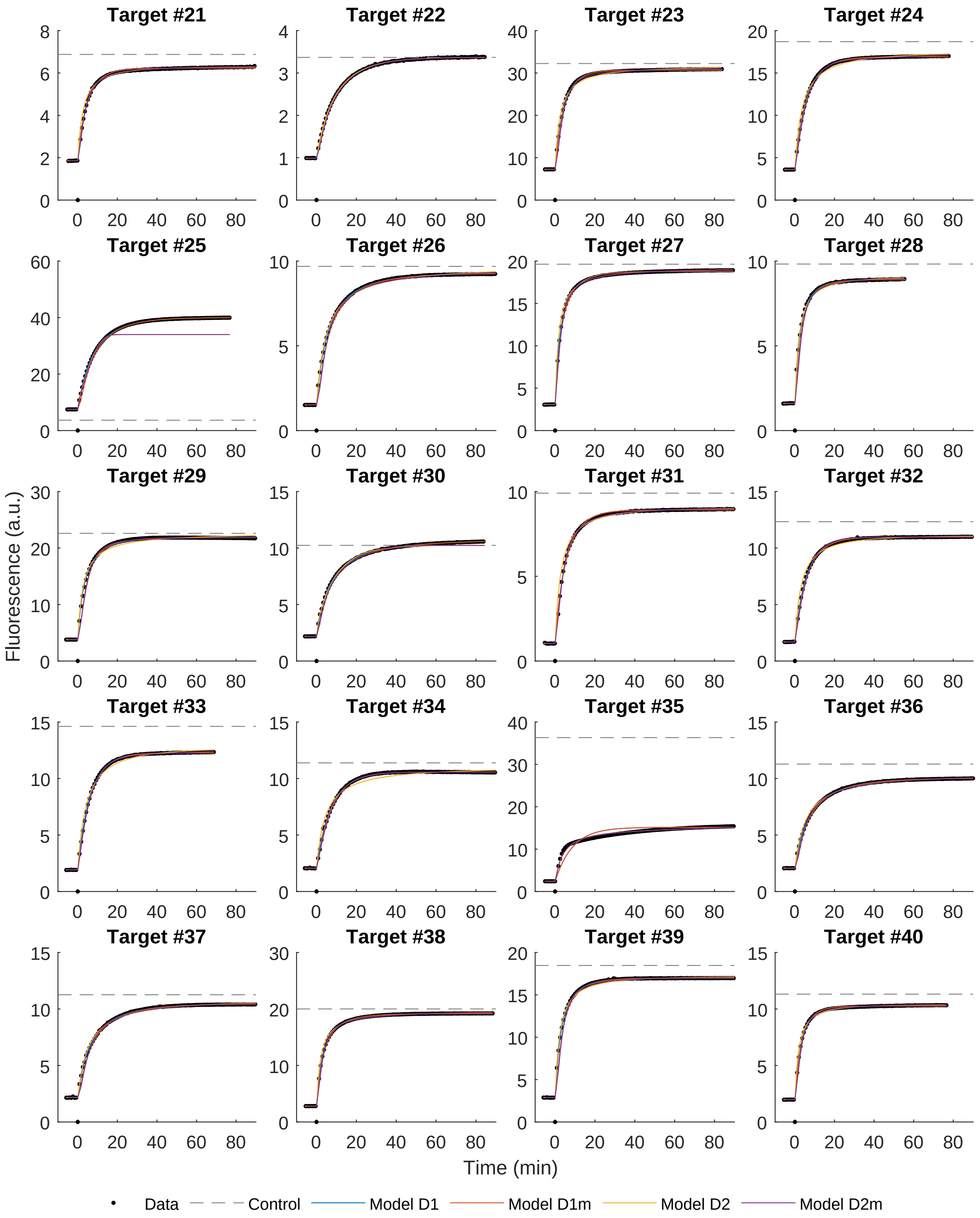

FIG. S10: **Fluorescence data and best-fit traces.** Strand displacement experiments performed at 37 °C, target sequences 21-40.

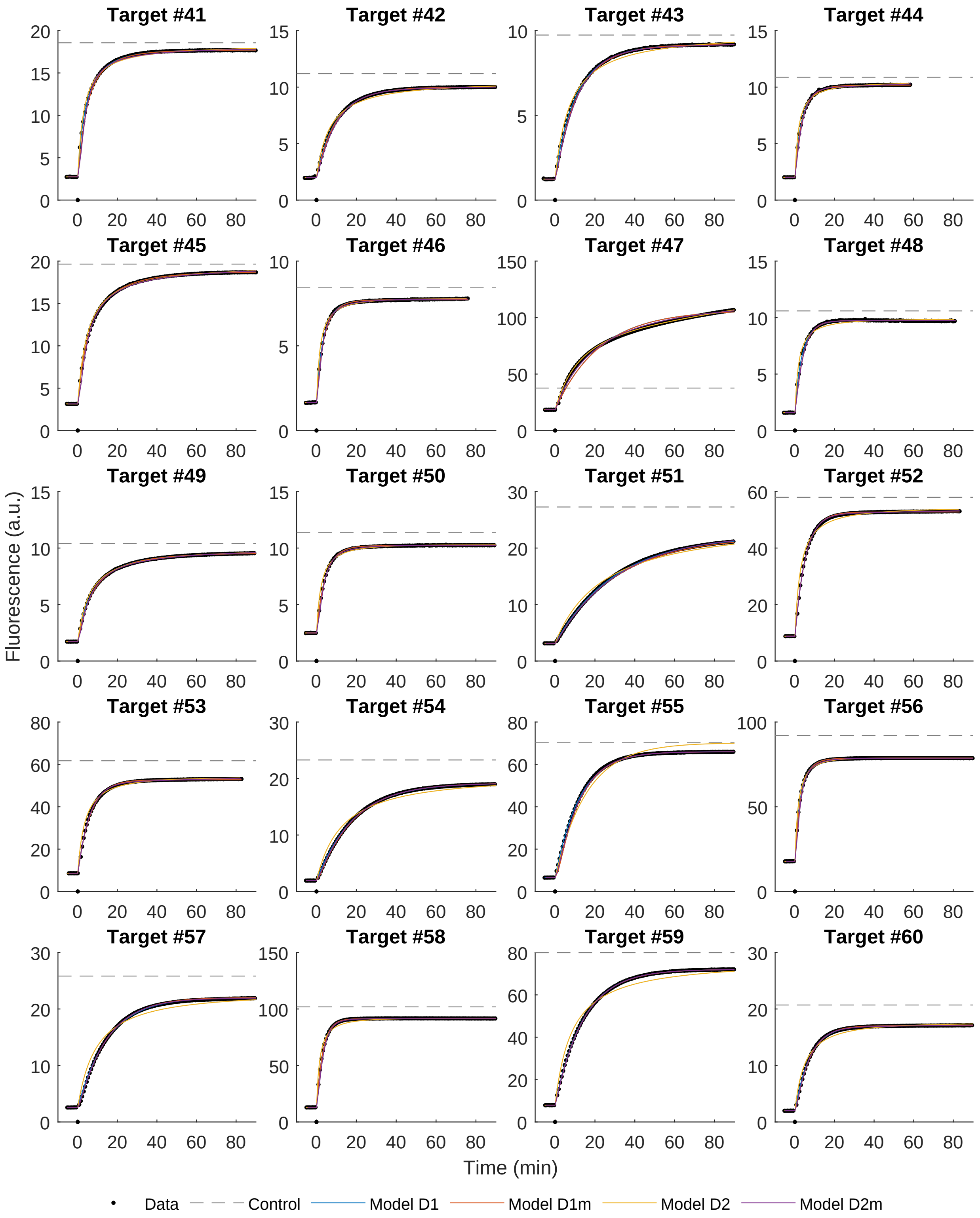

FIG. S11: **Fluorescence data and best-fit traces.** Strand displacement experiments performed at 37 °C, target sequences 41-60.

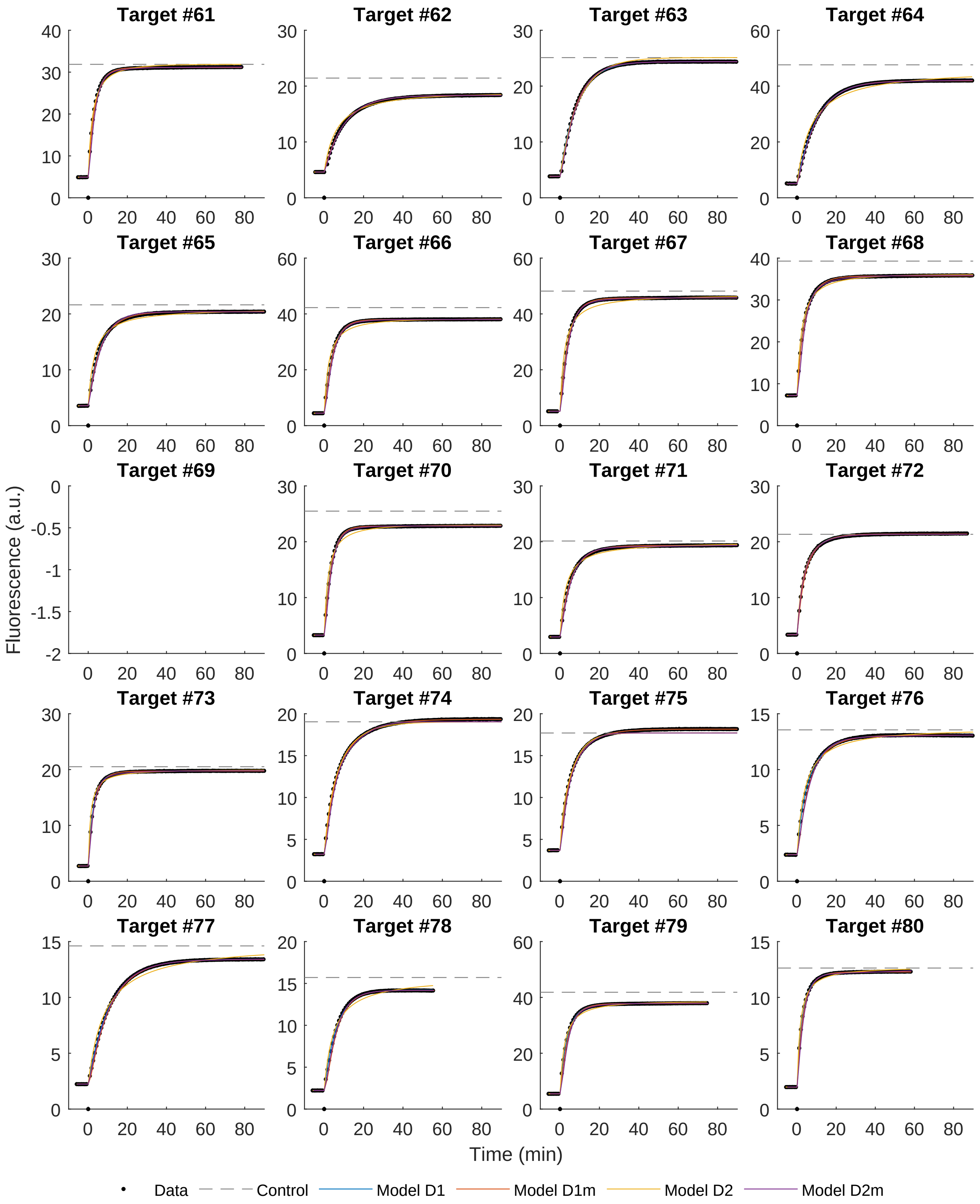

FIG. S12: **Fluorescence data and best-fit traces.** Strand displacement experiments performed at 37 °C, target sequences 61-80.

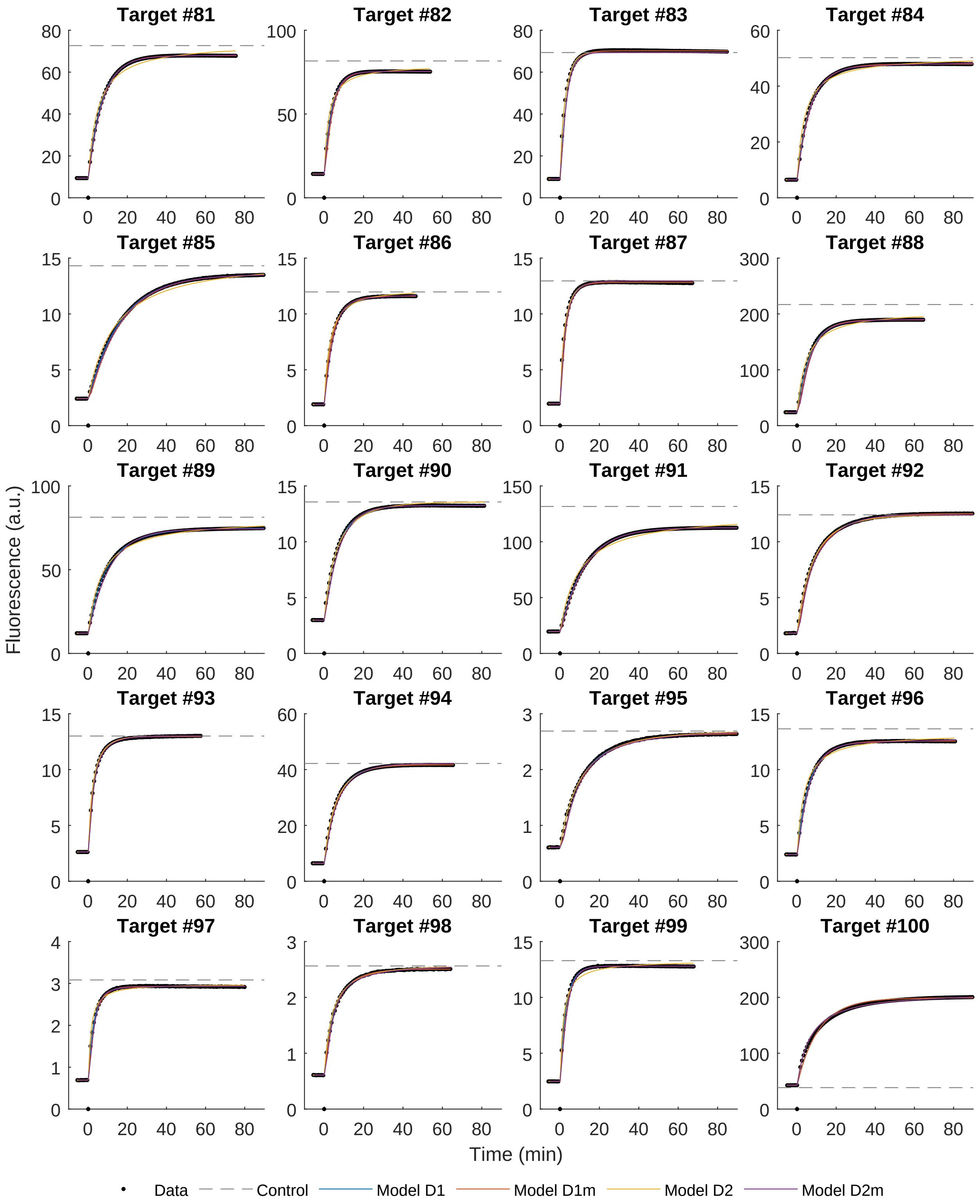

FIG. S13: **Fluorescence data and best-fit traces.** Strand displacement experiments performed at 37 °C, target sequences 81-100.

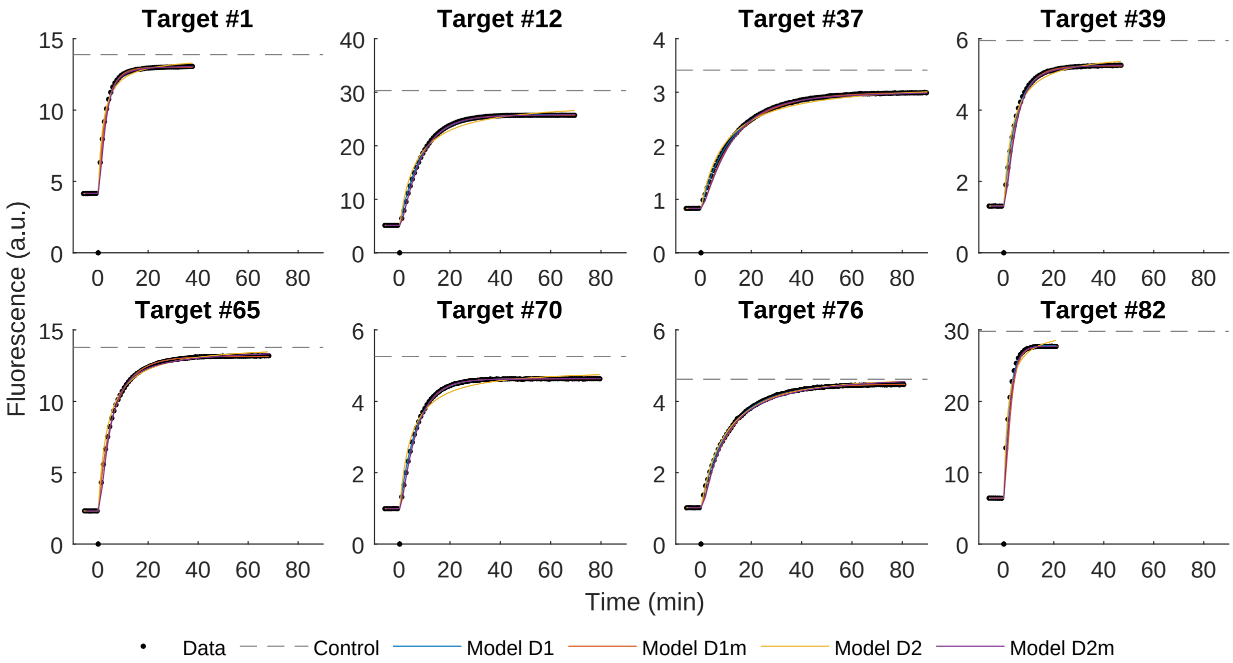

FIG. S14: **Fluorescence data and best-fit traces.** Strand displacement experiments performed at 46 °C.

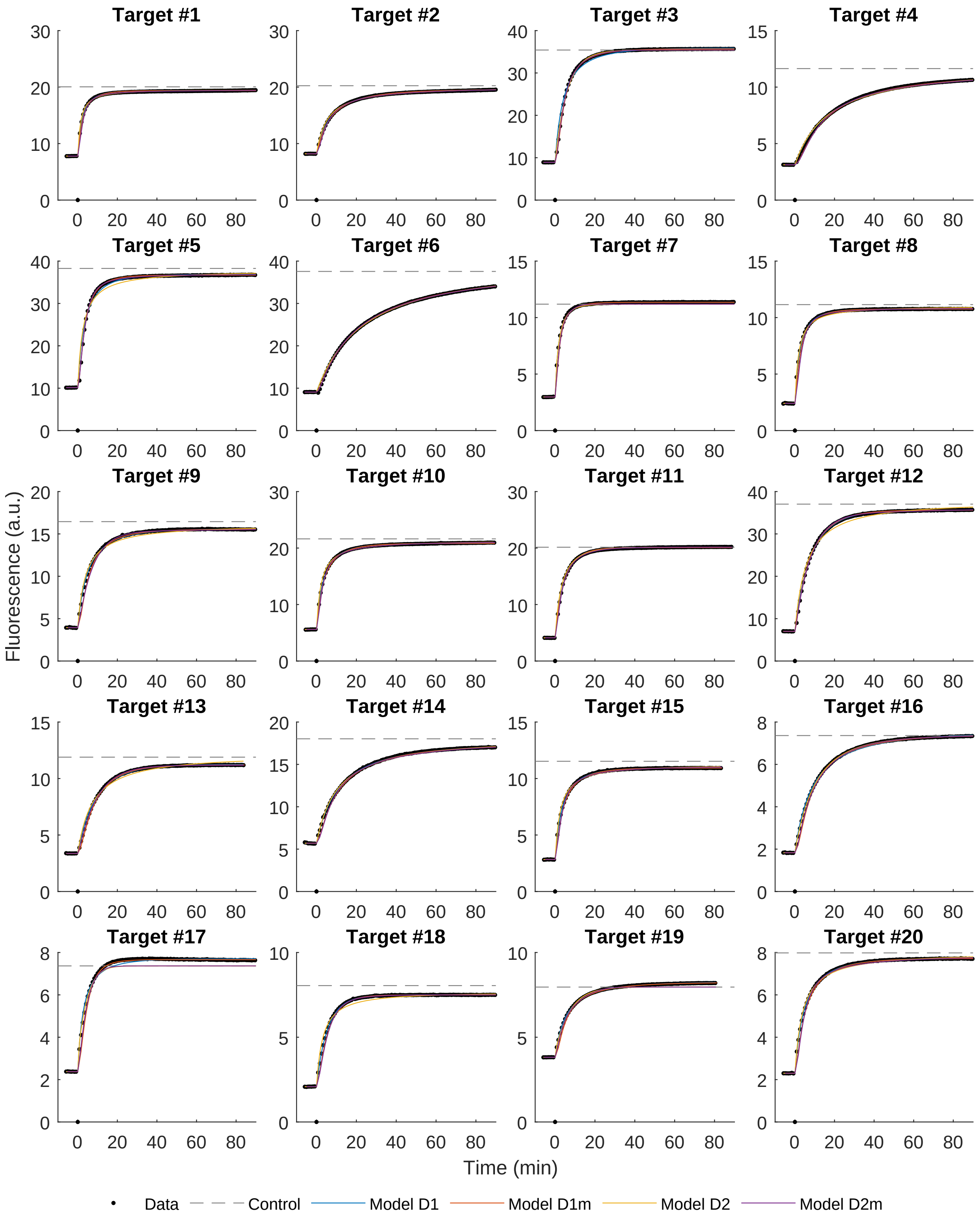

FIG. S15: **Fluorescence data and best-fit traces.** Strand displacement experiments performed at 55 °C, target sequences 1-20.

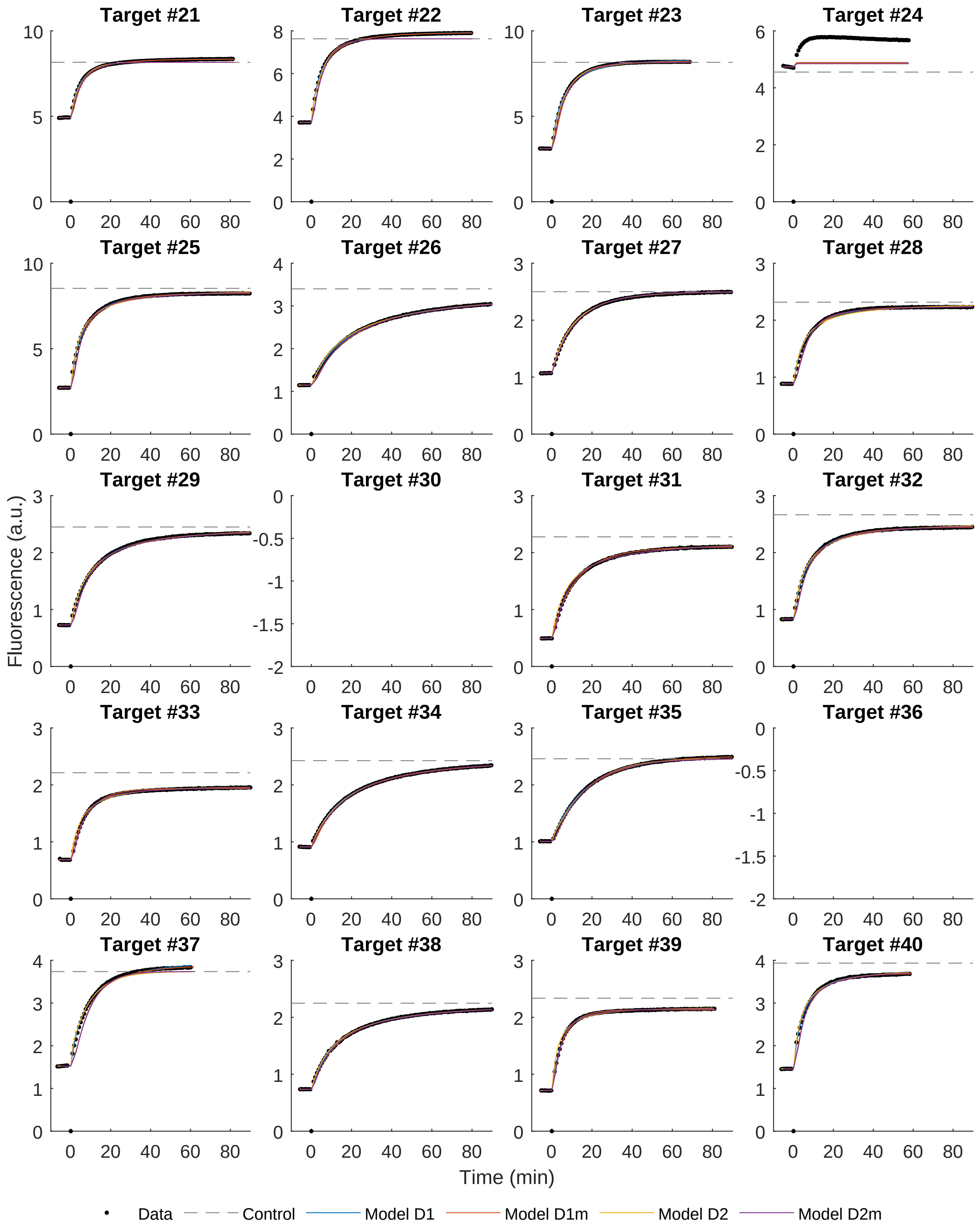

FIG. S16: **Fluorescence data and best-fit traces.** Strand displacement experiments performed at 55 °C, target sequences 21-40.

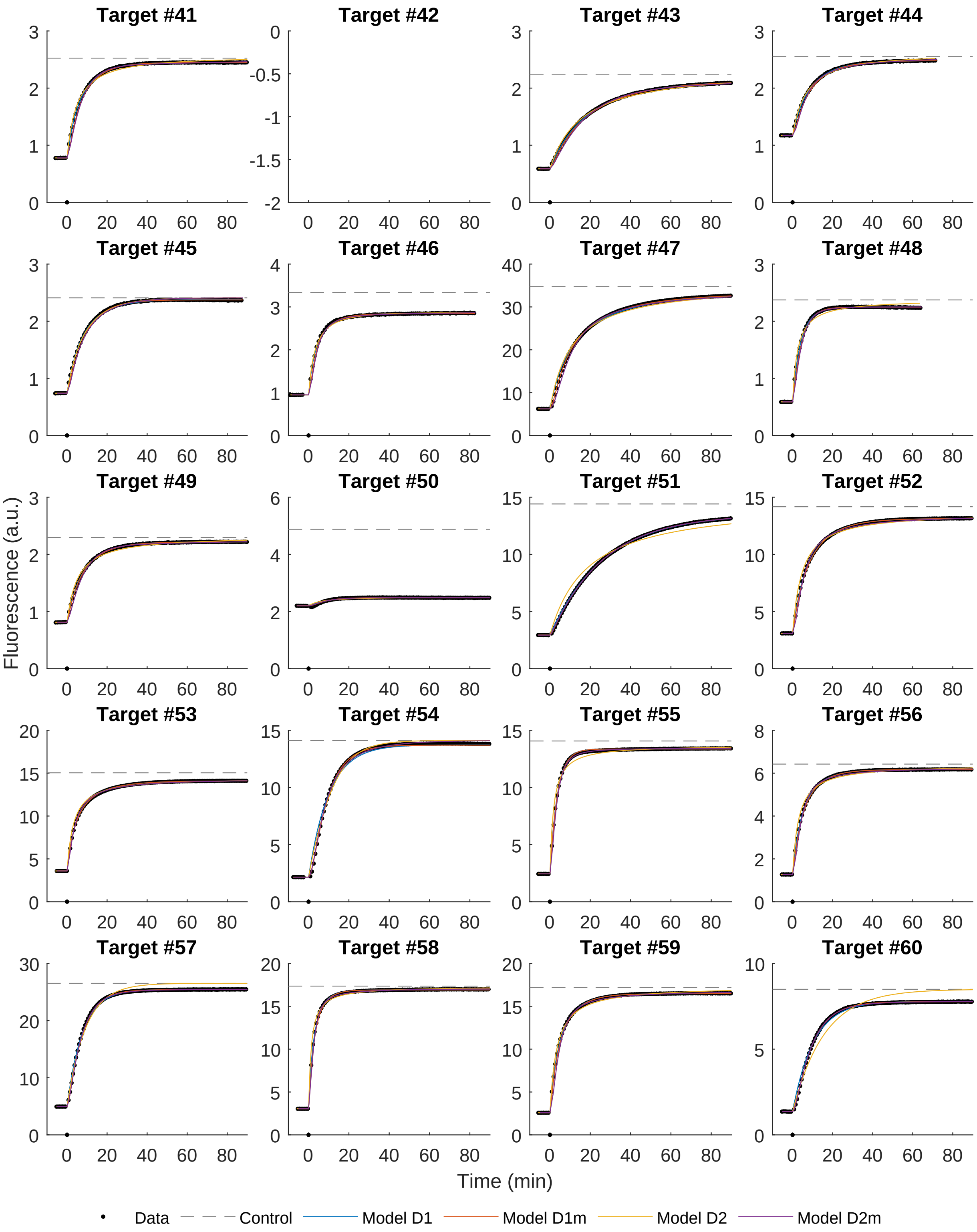

FIG. S17: **Fluorescence data and best-fit traces.** Strand displacement experiments performed at 55 °C, target sequences 41-60.

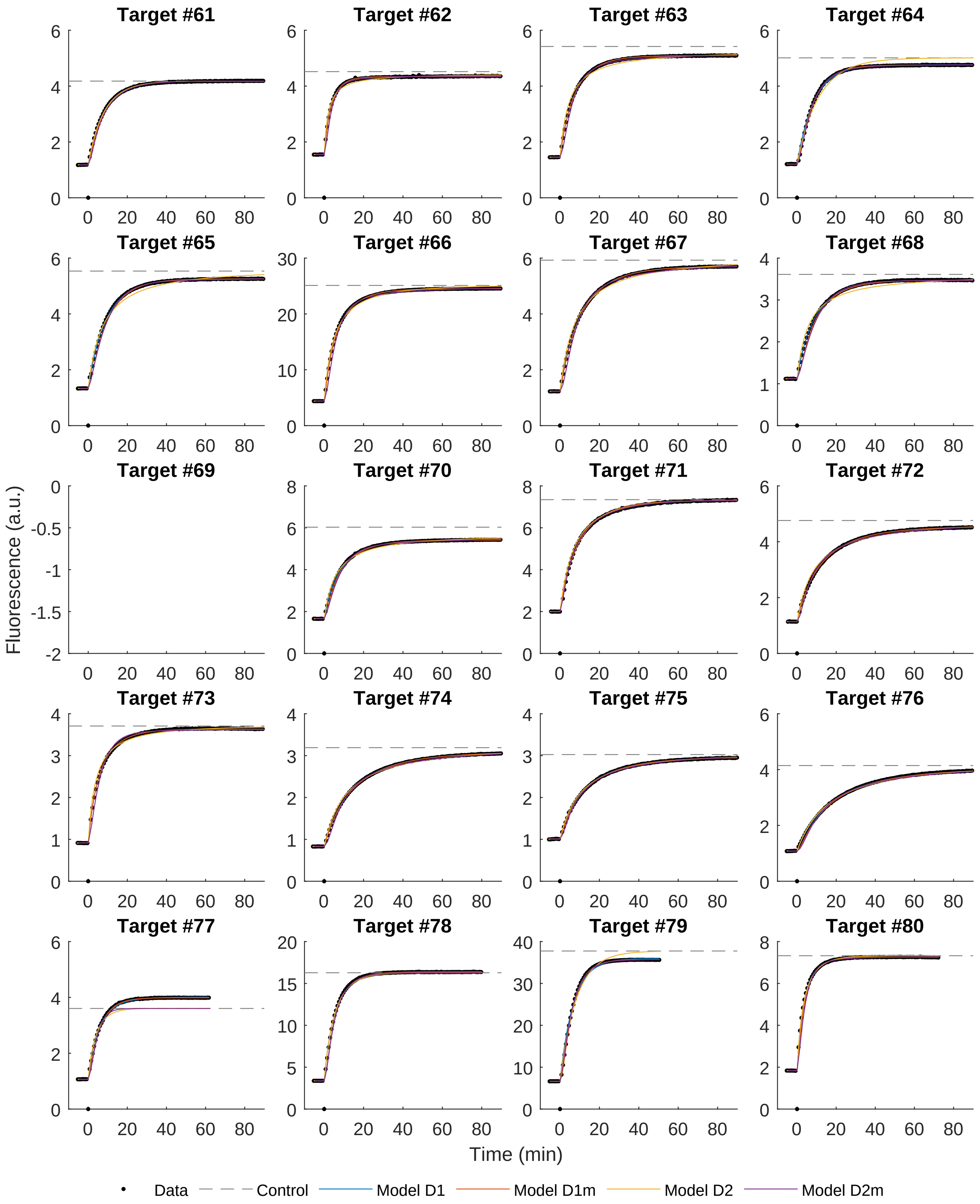

FIG. S18: **Fluorescence data and best-fit traces.** Strand displacement experiments performed at 55 °C, target sequences 61-80.

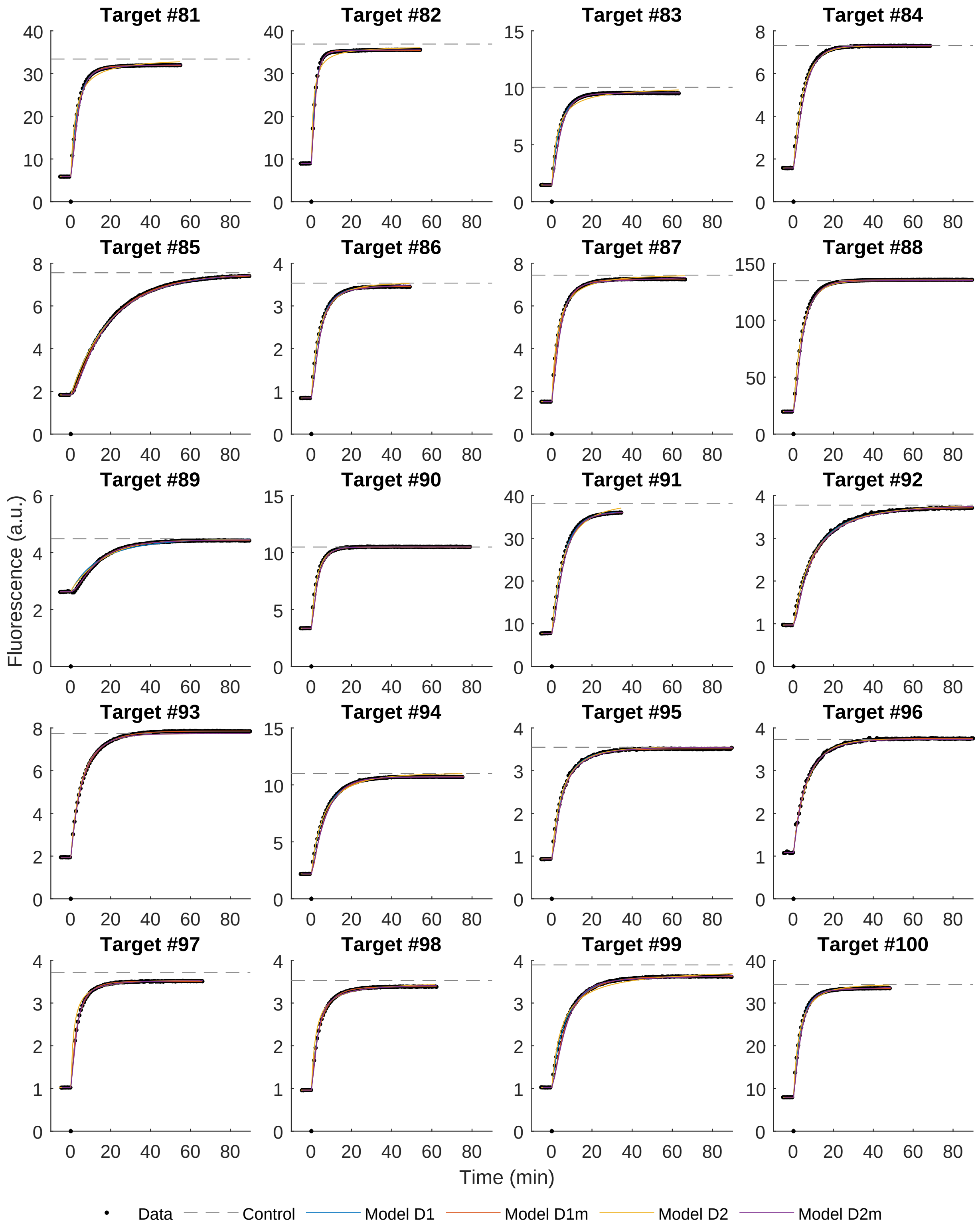

FIG. S19: **Fluorescence data and best-fit traces.** Strand displacement experiments performed at 55 °C, target sequences 81-100.

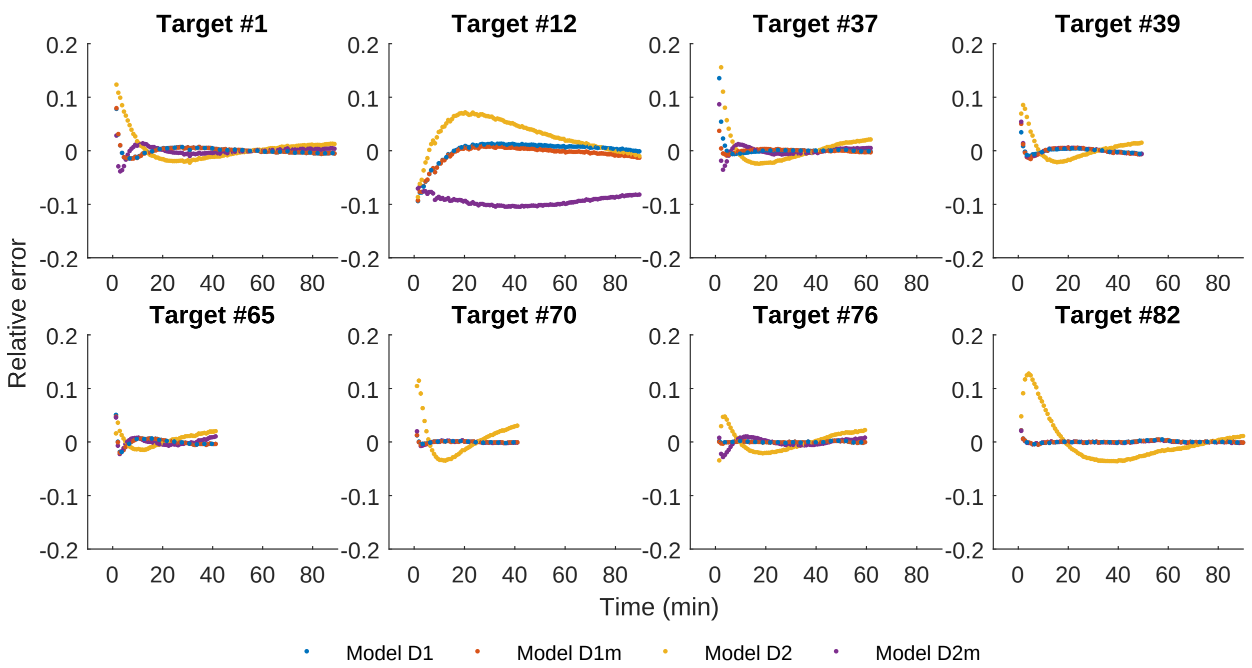

FIG. S20: **Relative errors of best-fit simulations.** Strand displacement experiments performed at 28 °C.

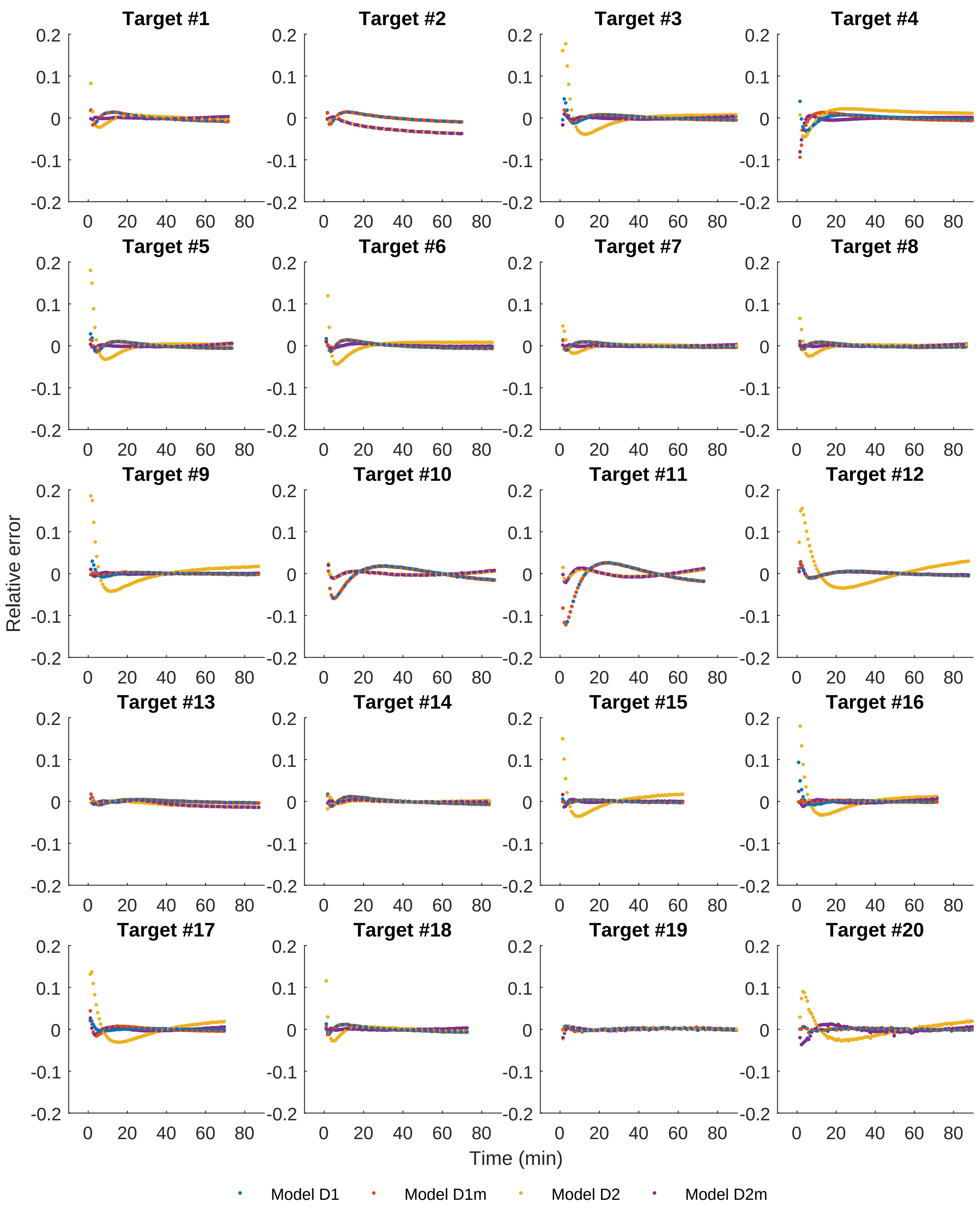

FIG. S21: **Relative errors of best-fit simulations.** Strand displacement experiments performed at 37 °C, target sequences 1-20.

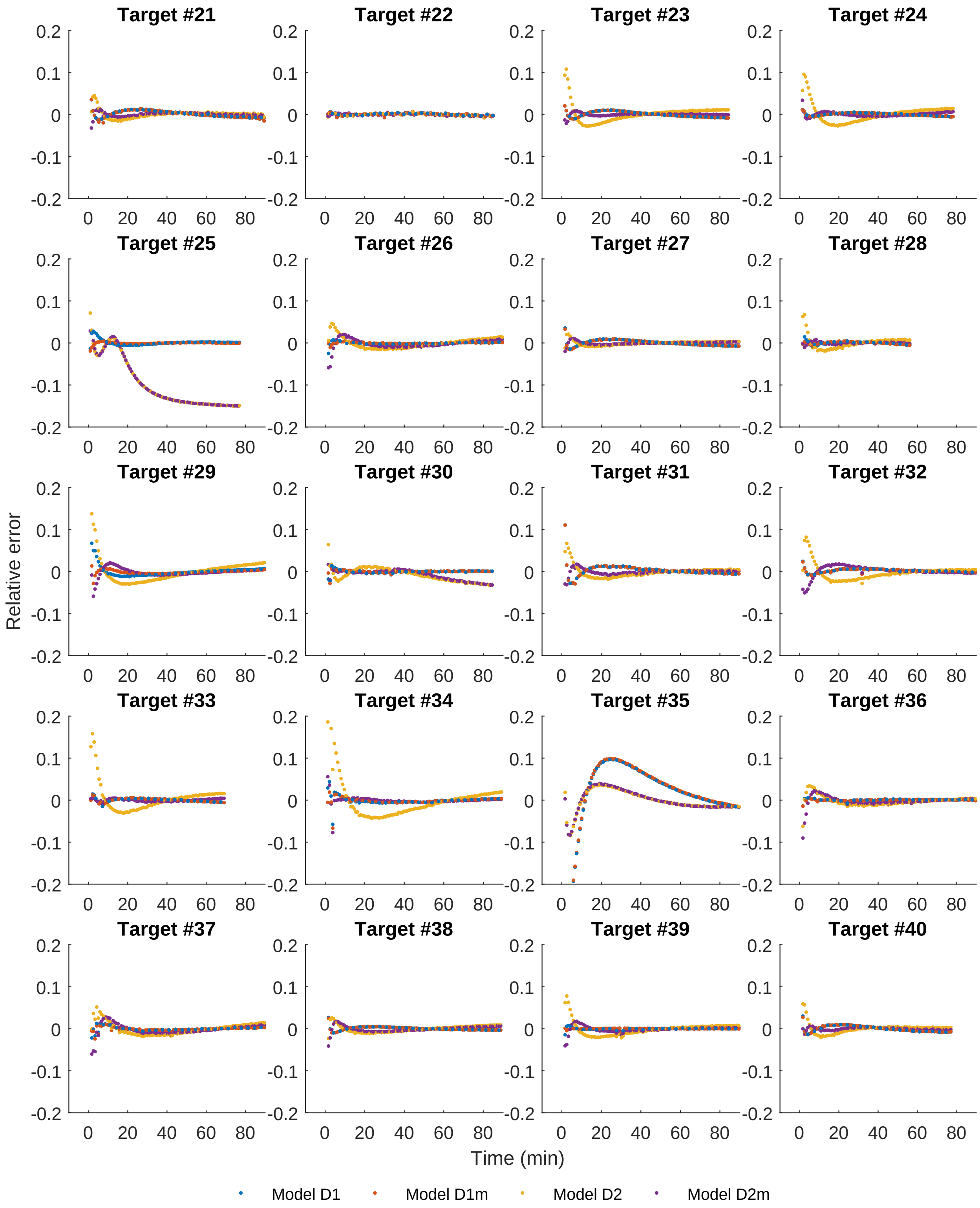

FIG. S22: **Relative errors of best-fit simulations.** Strand displacement experiments performed at 37 °C, target sequences 21-40.

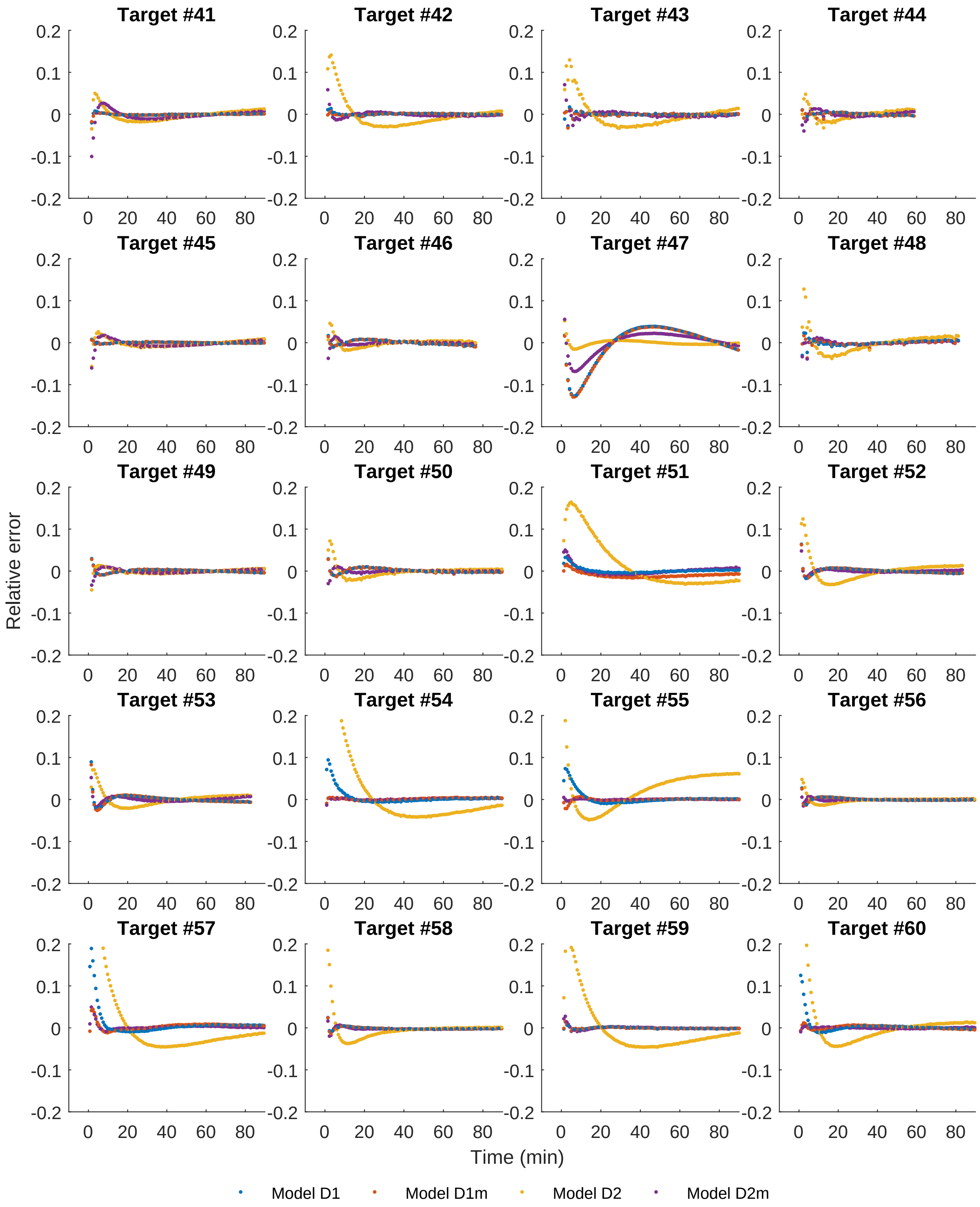

FIG. S23: **Relative errors of best-fit simulations.** Strand displacement experiments performed at 37 °C, target sequences 41-60.

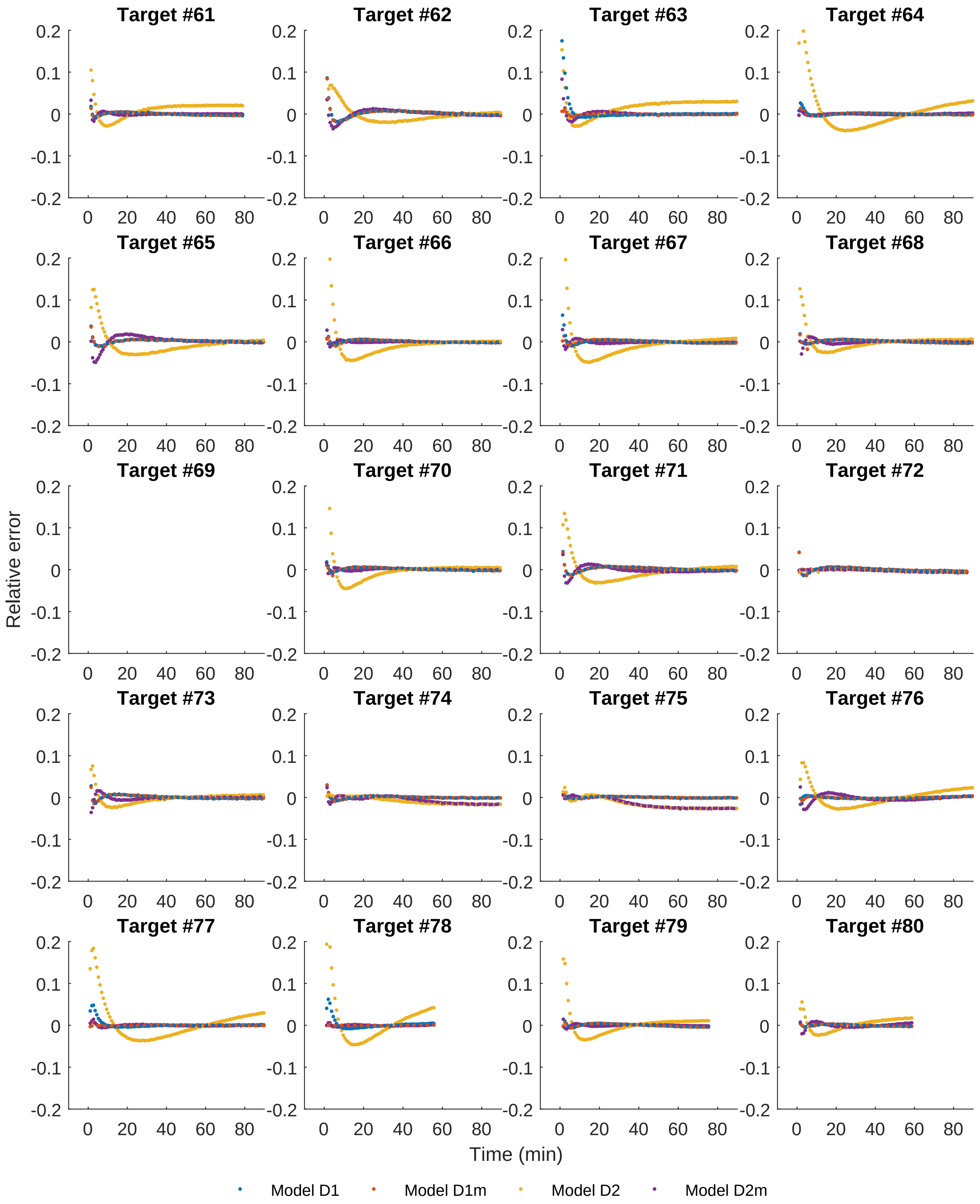

FIG. S24: **Relative errors of best-fit simulations.** Strand displacement experiments performed at 37 °C, target sequences 61-80.

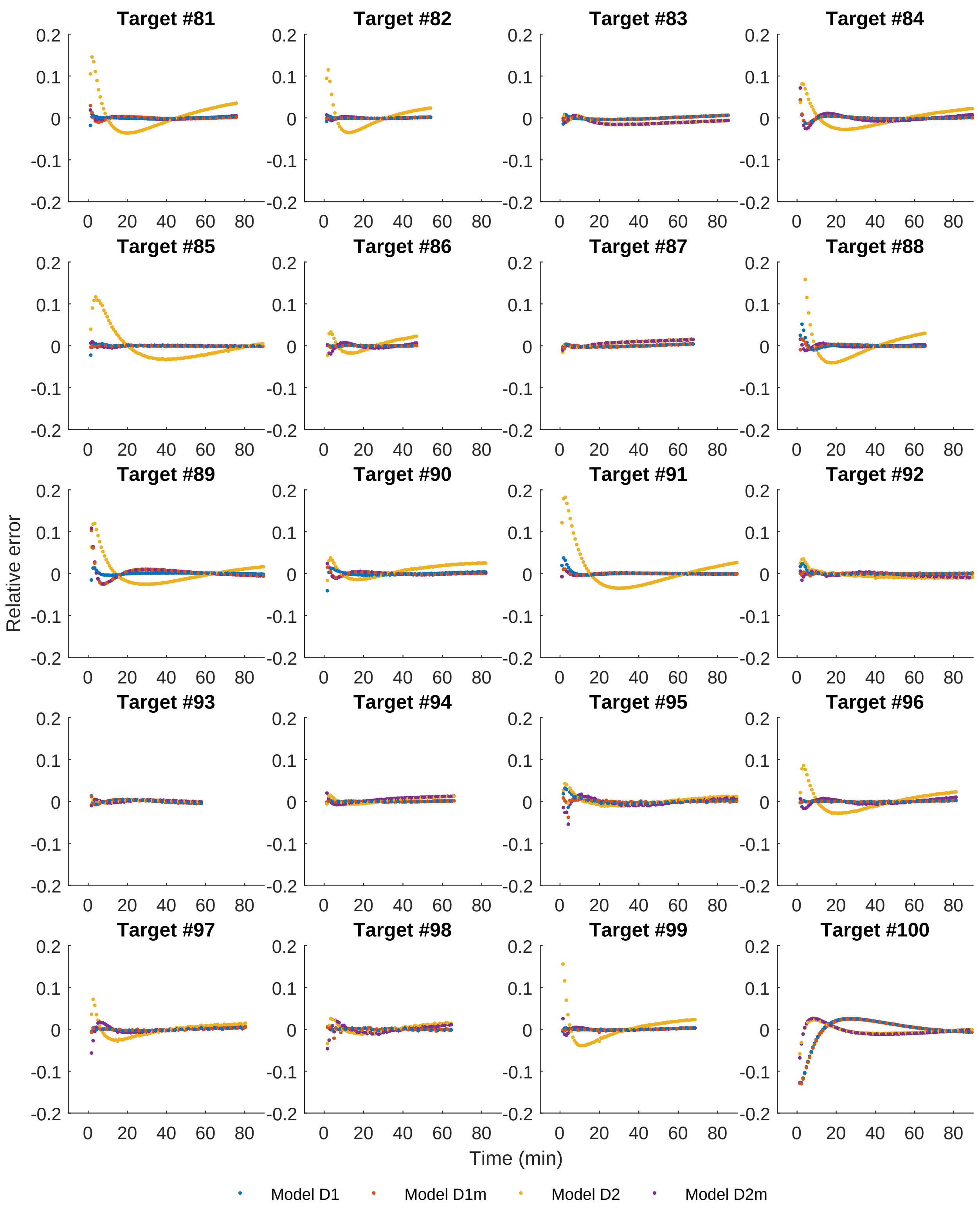

FIG. S25: **Relative errors of best-fit simulations.** Strand displacement experiments performed at 37 °C, target sequences 81-100.

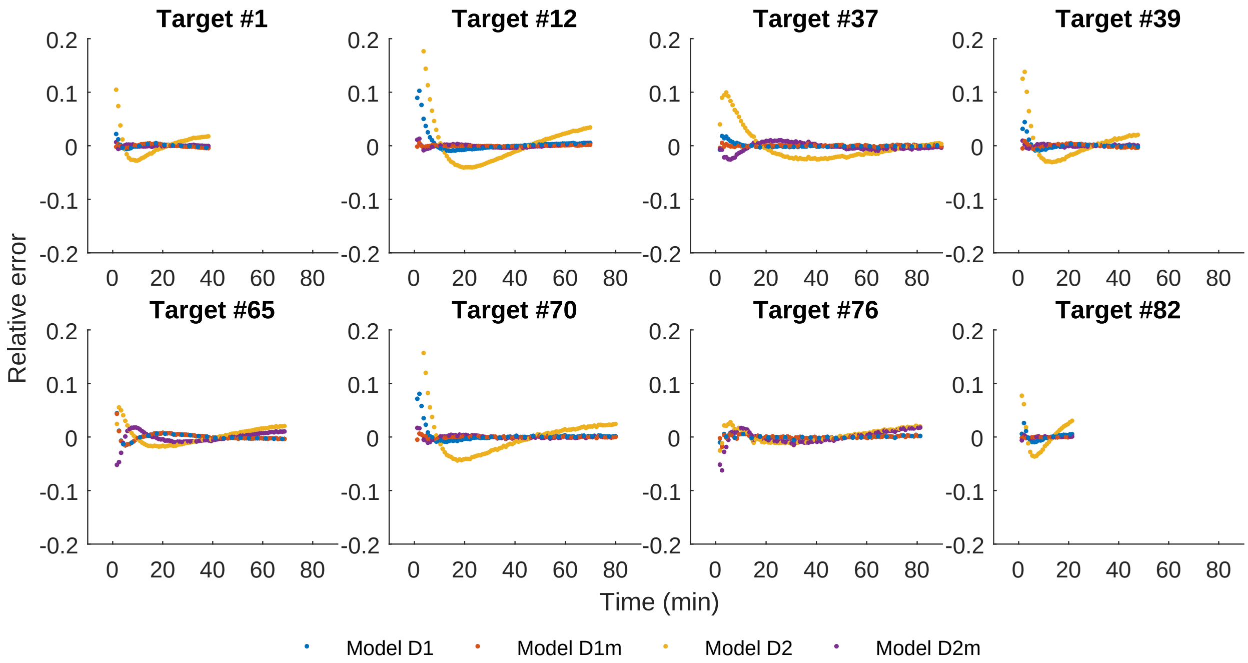

FIG. S26: **Relative errors of best-fit simulations.** Strand displacement experiments performed at 46 °C.

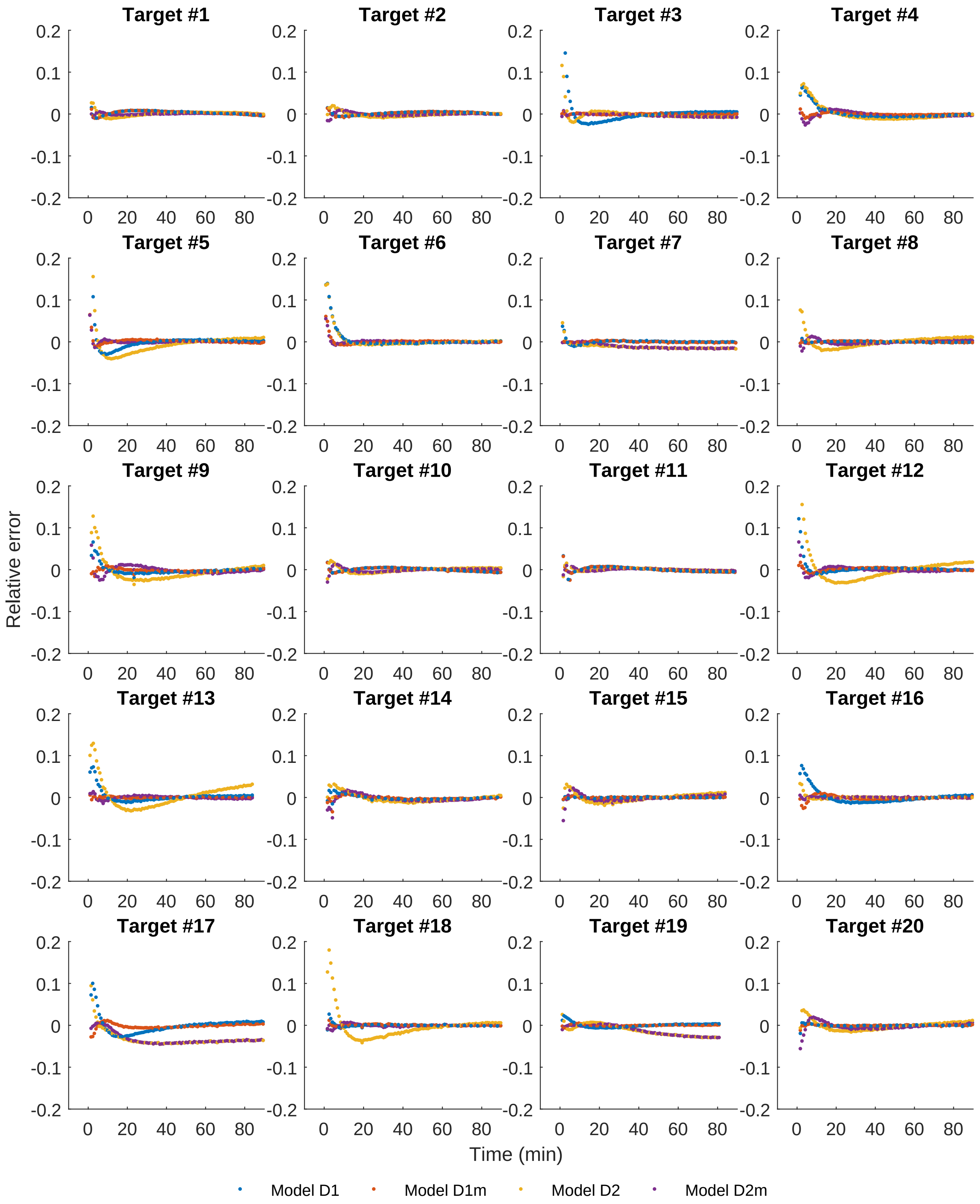

FIG. S27: **Relative errors of best-fit simulations.** Strand displacement experiments performed at 55 °C, target sequences 1-20.

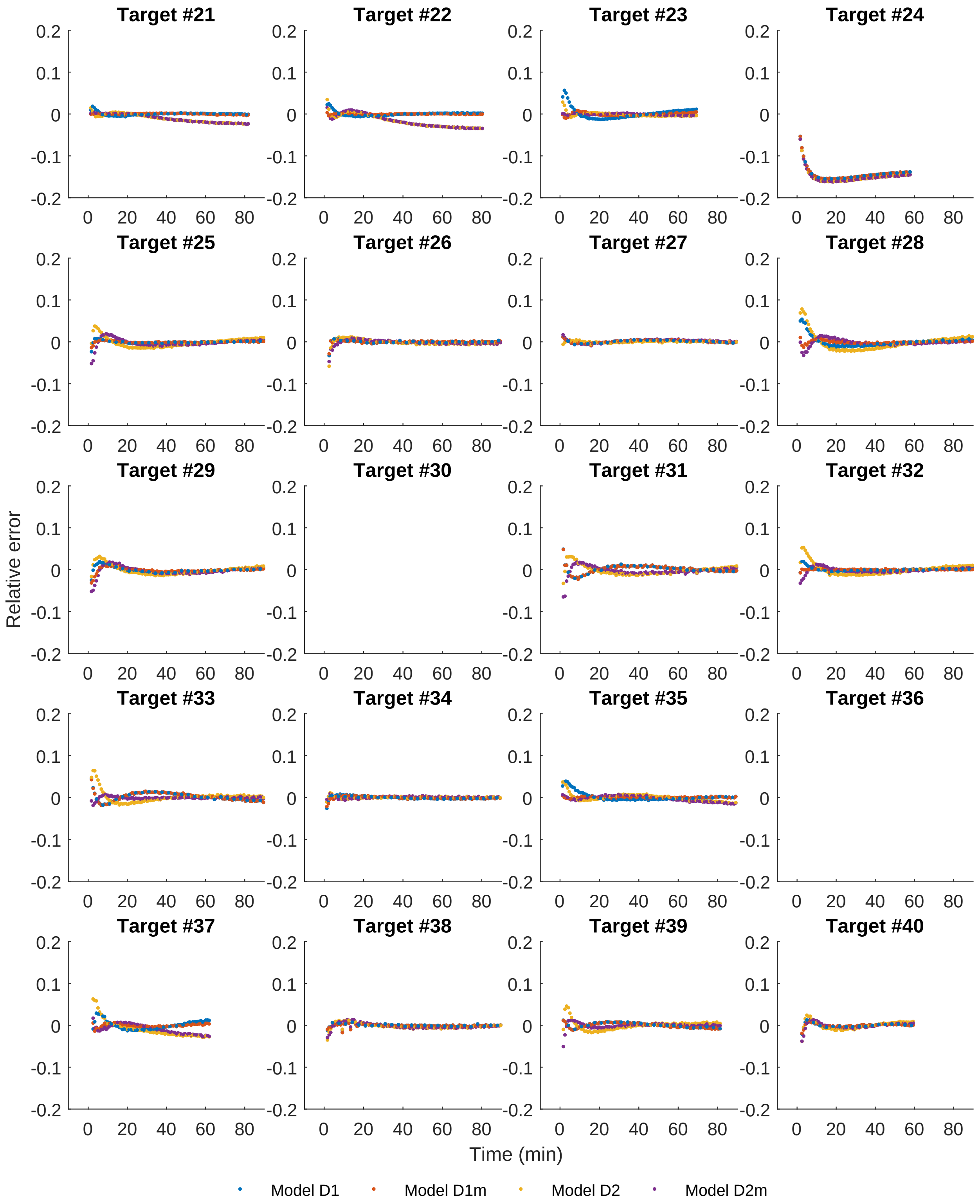

FIG. S28: **Relative errors of best-fit simulations.** Strand displacement experiments performed at 55 °C, target sequences 21-40.

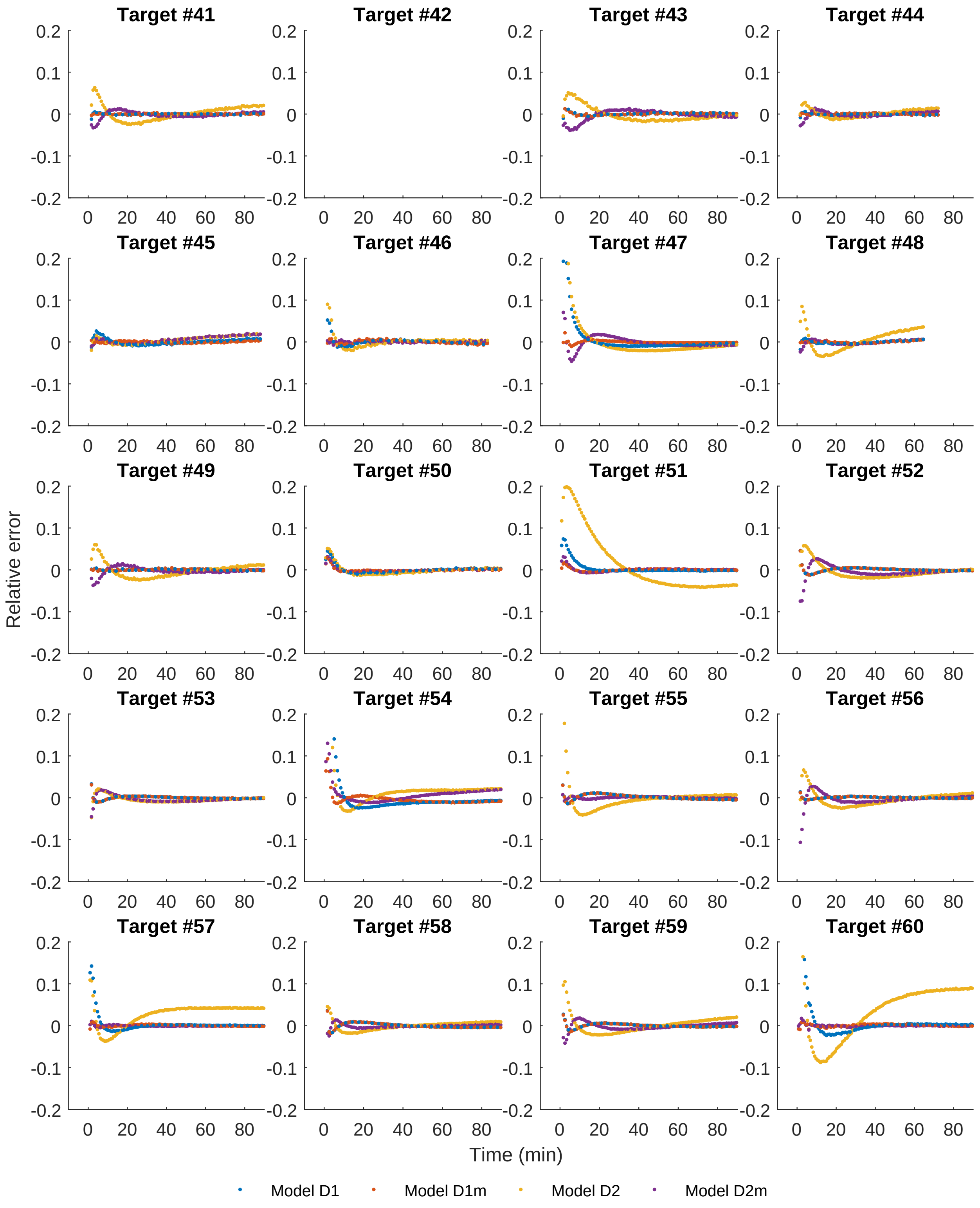

FIG. S29: **Relative errors of best-fit simulations.** Strand displacement experiments performed at 55 °C, target sequences 41-60.

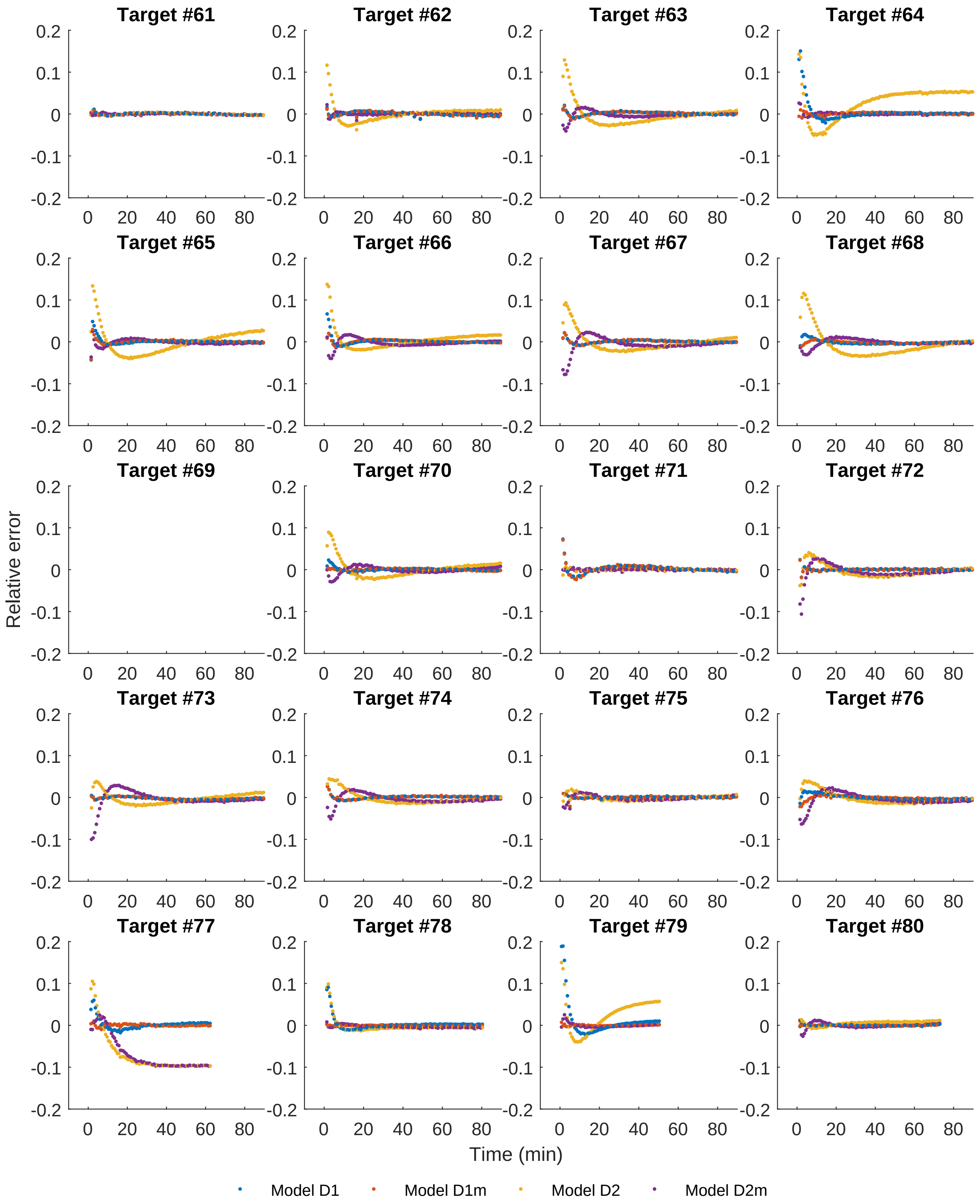

FIG. S30: **Relative errors of best-fit simulations.** Strand displacement experiments performed at 55 °C, target sequences 61-80.

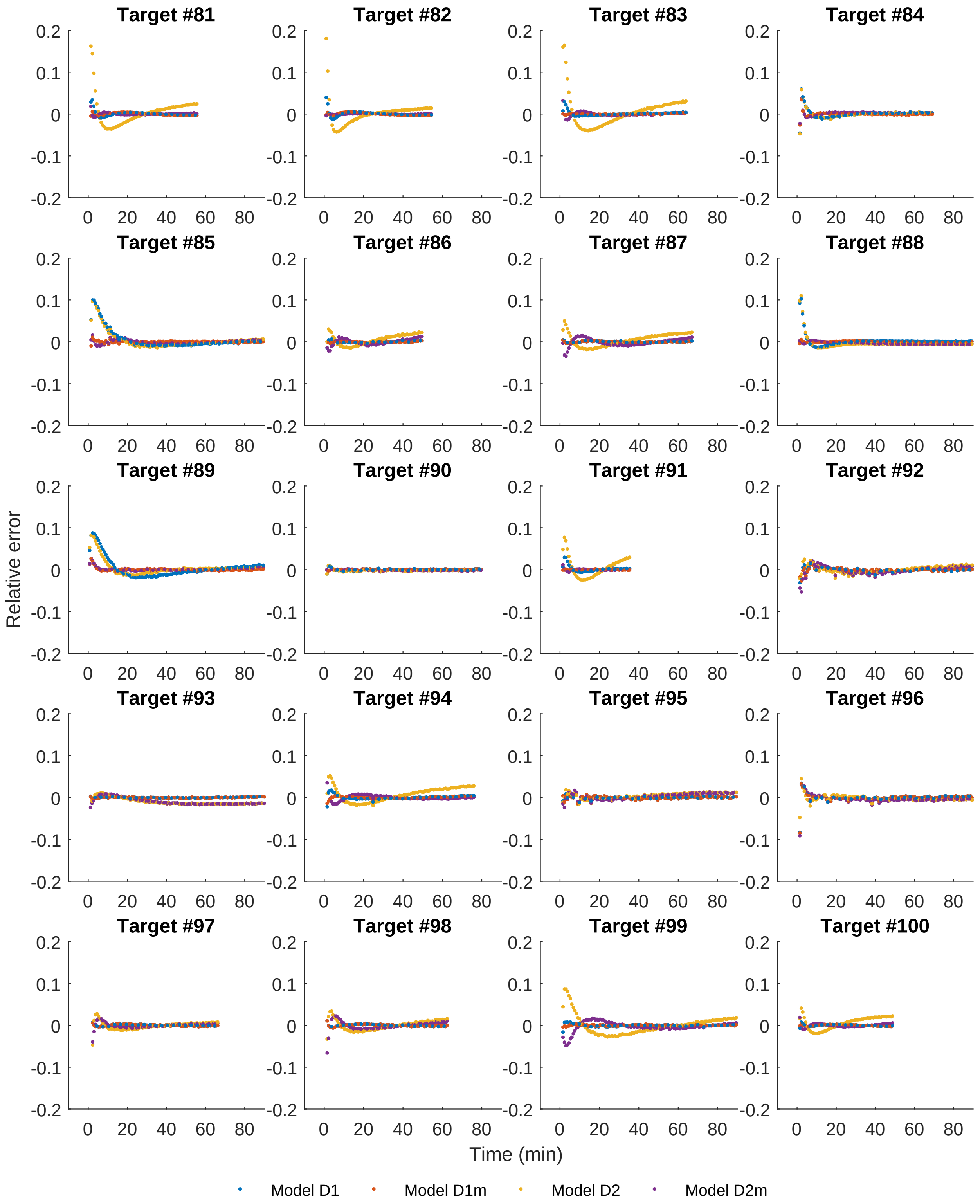

FIG. S31: **Relative errors of best-fit simulations.** Strand displacement experiments performed at 55 °C, target sequences 81-100.

Supplementary Note 3. Additional Experiments

Comparison between DLM and naive model. The naive model is defined as using the average of observed data in the training set to predict all the sequences in the validation set. Taking the 20-fold cross-validation of Human SNP panel for example, for each training set the average of observed $\log_{10}(\text{depth})$ is used to predict the corresponding validation set, and the naive predictions of 20 validation sets are compared against the predictions of DLM.

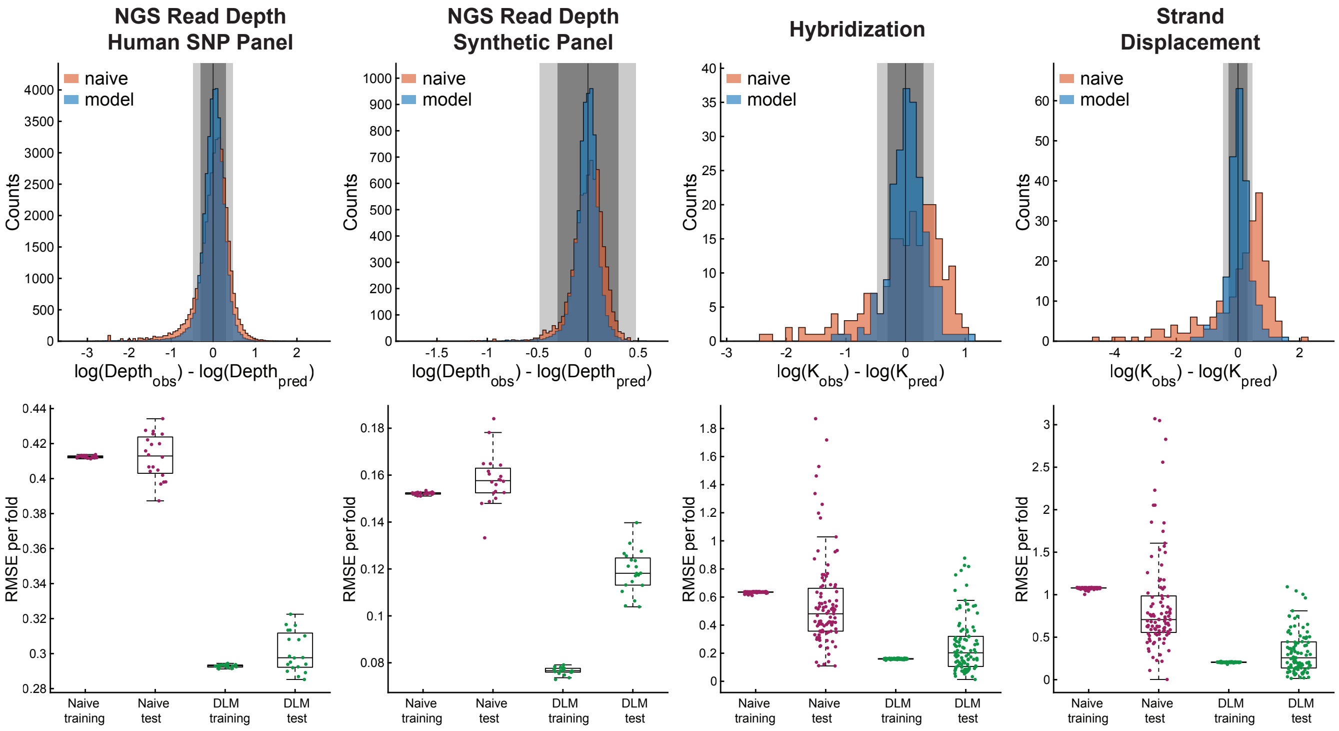

FIG. S32: The first row shows the distribution of prediction error of both DLM and naive model, in which predictions of all validations sets are combined together. Dark gray shading marks the zones where the predicted and the observed read depth agreed to within a factor of 2; light gray shows agreement to within factor of 3. The second row shows the summary of root-mean-square error (RMSE) in the same format as in main text Fig. 2c. Each point corresponds to the prediction results for one of the 20 (NGS panel) or 100 (Hybridization or strand displacement) validation classes. In box-whisker plots, the central mark indicates the median, and the bottom and top edges of the box indicate the 25th and 75th percentiles, respectively. The maximum whisker lengths are specified as 1.5 times the interquartile range.

Comparison between DLM and linear regression model. The linear regression model fits the observed $\log_{10}(\text{Depth})$ or observed $\log_{10}(\text{k})$ against the four global features with a linear function,

$$\textbf{prediction} = \mathbf{a}_0 + \mathbf{a}_1 \cdot \Delta G^{\circ}(P) + \mathbf{a}_2 \cdot \Delta G^{\circ}(T) + \mathbf{a}_3 \cdot \Delta G^{\circ}(TP) + \mathbf{a}_4 \cdot T \quad (3)$$

where $\mathbf{a}_0$ to $\mathbf{a}_4$ are model parameters that minimizes the mean squared error between the predicted and observed data. We also performed 20-fold cross-validation or 100-fold LOCO and the linear regression model was better than the naive model but worse than the DLM on all datasets.

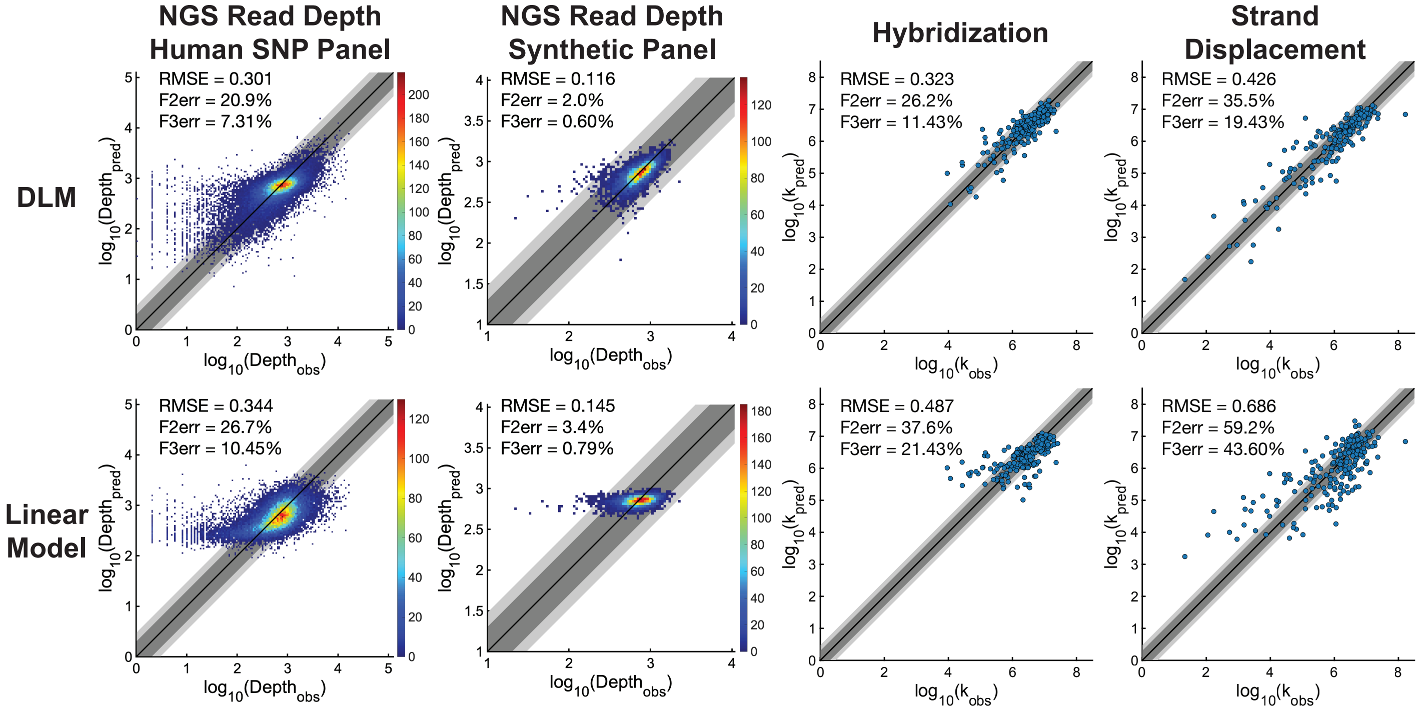

FIG. S33: The first row shows the predictions of the DLM and the second row shows the predictions of the linear regression model. Each panel shows the aggregated results of all the validation classes (20 classes for NGS dataset, 100 classes for kinetics datasets) Dark gray shading marks the zones where the predicted and the observed read depth agreed to within a factor of 2; light gray shows agreement to within factor of 3.

Training and predicting across datasets. Since the total reads of a NGS panel would affect the actual read depth (e.g., 5M vs. 10M for the same 1000 probes), we first normalized the read depth of each probe by dividing the average read depth (denoted as avg_depth) of that NGS panel, so that the average read depth changed to one. Downstream analysis was the same as the training and predicting within the same dataset.

$$\log_{10}(NormDepth) = \log_{10}(Depth/avg\_depth) = \log_{10}(Depth) - \log_{10}(avg\_depth) \quad (4)$$

The mean and standard deviation of the train set were used to normalize the global features and rescale the predictions of the test set. Note that if a probe P is fed to a DLM trained on a NGS panel A, the prediction is the expected read depth of probe P in the context of panel A, which does not have to correlate with the observed read depth of probe P in panel B. Such correlation depends on the library preparation methods of panel A and B. For the DLM trained on the SNP panel, the Pearson correlation coefficient between the predicted and observed $\log_{10}(\text{NormDepth})$ of the lncRNA panel is 0.728, while the Pearson correlation coefficient of the synthetic panel is only 0.319 (Fig. S34). This is because the SNP panel and the lncRNA panel use the same library preparation method. We noticed that the predicted read depth of probes in the synthetic panel is above average (zero $\log_{10}(\text{NormDepth})$ correspond to average read depth), which might be because those probes are specially designed to have high hybridization yield instead of chosen from human genome.

One could reasonably hypothesize that a probe with higher hybridization rate constant would yield higher NGS read depth. Although the true values of hybridization rate constants for our NGS probes are not known, we could predict the rate constants of those probes with the DLM trained simultaneously on our hybridization (HYB) and strand displacement (DSP) dataset. On the contrary, we could predict the read depth of the probes in the HYB or

DSP dataset with a DLM trained on one of the NGS dataset. However, we did not see a significant correlation in either case. There should be two possible explanations, 1) the wide difference of probe length between NGS panels and kinetics experiments makes the predictions of the hybridization rate constant of NGS probes inaccurate, 2) there is only a weak correlation between the NGS read depth and the hybridization rate constant of a certain probe. Note that the features of the DSP dataset are calculated not only based on the sequences of the probes, but also the sequences of the protectors that greatly reduce open base probabilities, resulting in low predicted read depth of the DSP dataset.

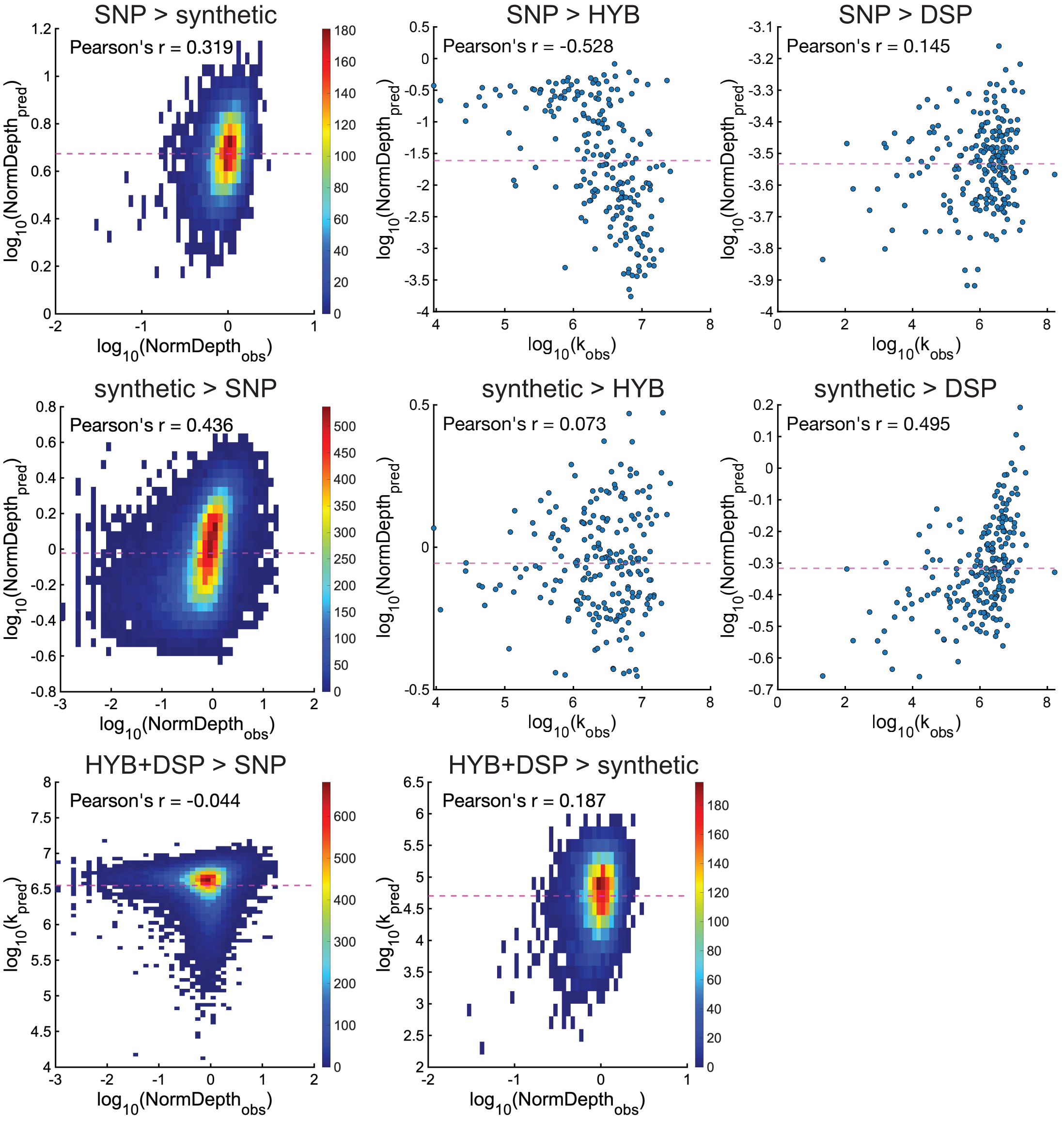

FIG. S34: Each figure shows the predictions of test sets. For example, SNP > synthetic denotes training on the SNP dataset and test on the synthetic dataset. Dashed magenta lines show the average of predictions.

We further applied the DLM trained on the SNP panel to two commercial NGS panels with public probe sequences: xGen Exome Research Panel v2 (Integrated DNA Technologies), abbreviated as the exome panel, and xGen Acute Myeloid Leukemia Cancer Panel (Integrated DNA Technologies), abbreviated as the AML panel. These two panels have the same library preparation method (120nt probe length and 65°C hybridization temperature) but different probe sequences: the exome panel has 415,115 probes covering human whole exome and the AML panel has 11,731 probes targeting more than 260 human genes. The prediction results are shown in Fig. S35. The exome panel and the AML panel have higher median predicted read depth than the SNP panel and the lncRNA panel, which might be attributed to longer probe length (120nt vs. 80nt). The synthetic panel has the highest median predicted read depth and the lowest variation since its probe sequences are artificially designed for hybridization.

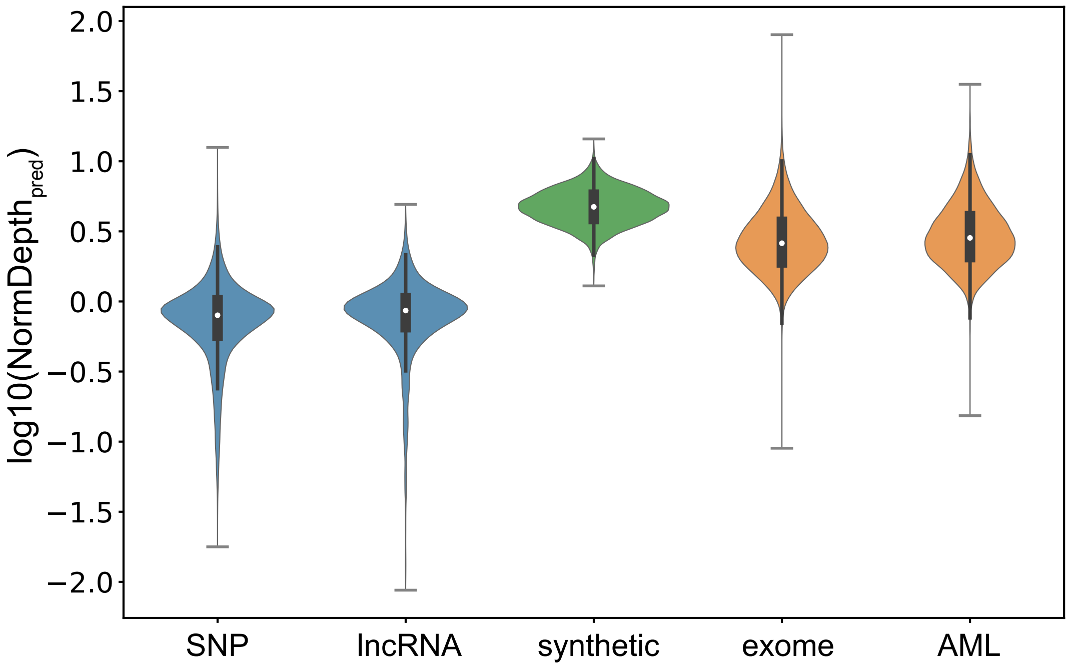

FIG. S35: The DLM trained on the SNP panel is applied to commercial panels with public probe sequences. SNP: human single nucleotide polymorphisms panel, 39,145 probes. lncRNA: human long non-coding panel, 2000 probes. synthetic: artificially designed synthetic sequences for information storage, 7,373 probes. exome: xGen Exome Research Panel v2, 415,115 probes. AML: xGen Acute Myeloid Leukemia Cancer Panel, 11,731 probes. Color code represents library preparation method. In box-whisker plots, the central mark indicates the median, and the bottom and top edges of the box indicate the 25th and 75th percentiles, respectively. The maximum whisker lengths are specified as 1.5 times the interquartile range. Maxima and minima are shown in gray lines.

DLM Reproducibility on Predicting NGS Read Depth of Human Genomic DNA Panel. We performed 15 replicate experiments on DLM predicting human genomic DNA panel NGS read depth. For each replicate, we split individual and random data, as well as independent initial values of RNN nodes of each fold. In Fig. S36, we plotted all 15 replicates in the same format as in main text Fig. 2d, where we aggregated 20-fold cross validation results in the same figure and then evaluated them based on F2acc, F3acc and RMSE values.

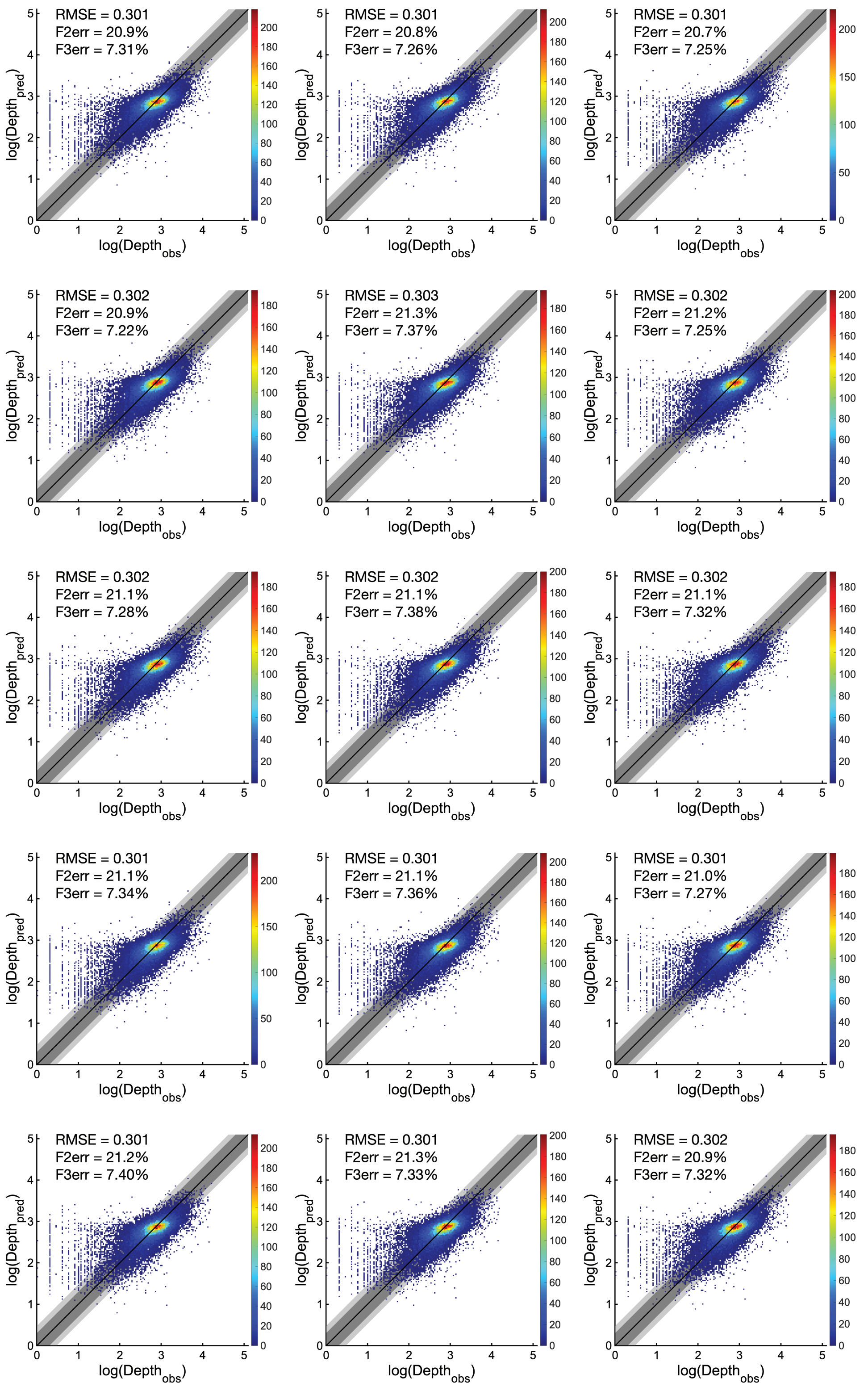

FIG. S36: 15 replicates of DLM predicting NGS read depth of human genomic DNA panel. N= 38040 for all figures. Dark gray shading marks the zones where the predicted and the observed read depth agreed to within a factor of 2; light gray shows agreement to within factor of 3.

DLM Performance on Predicting Rate Constants using Different Training Set Sizes. In this manuscript, we primarily considered the leave-one-class-out (LOCO) predictions, in which the entire dataset except the predicted target DNA sequence was used to predict one target DNA sequence's rate constant at different temperatures. Here, we also tested the DLM for predicting strand displacement rate constants using only a fraction of the entire dataset as the training set, and observing the prediction performance on the remainder of the dataset as a test set. Fig. S37 shows sample DLM prediction results for training sets using between 10% and 90% of the overall dataset, and Fig. S38 shows a summary of the results of 10 different experiments selecting different subsets of the data as the training set. The prediction accuracy plateaus at near the usage of roughly 60% of the dataset as the training set. For very large training set sizes (e.g. 90%), there is higher variation in prediction accuracy, due to the stochasticity associated with smaller test sets. The observed results are generally consistent with our expectations, and supports our claims on the accuracy of DLM.

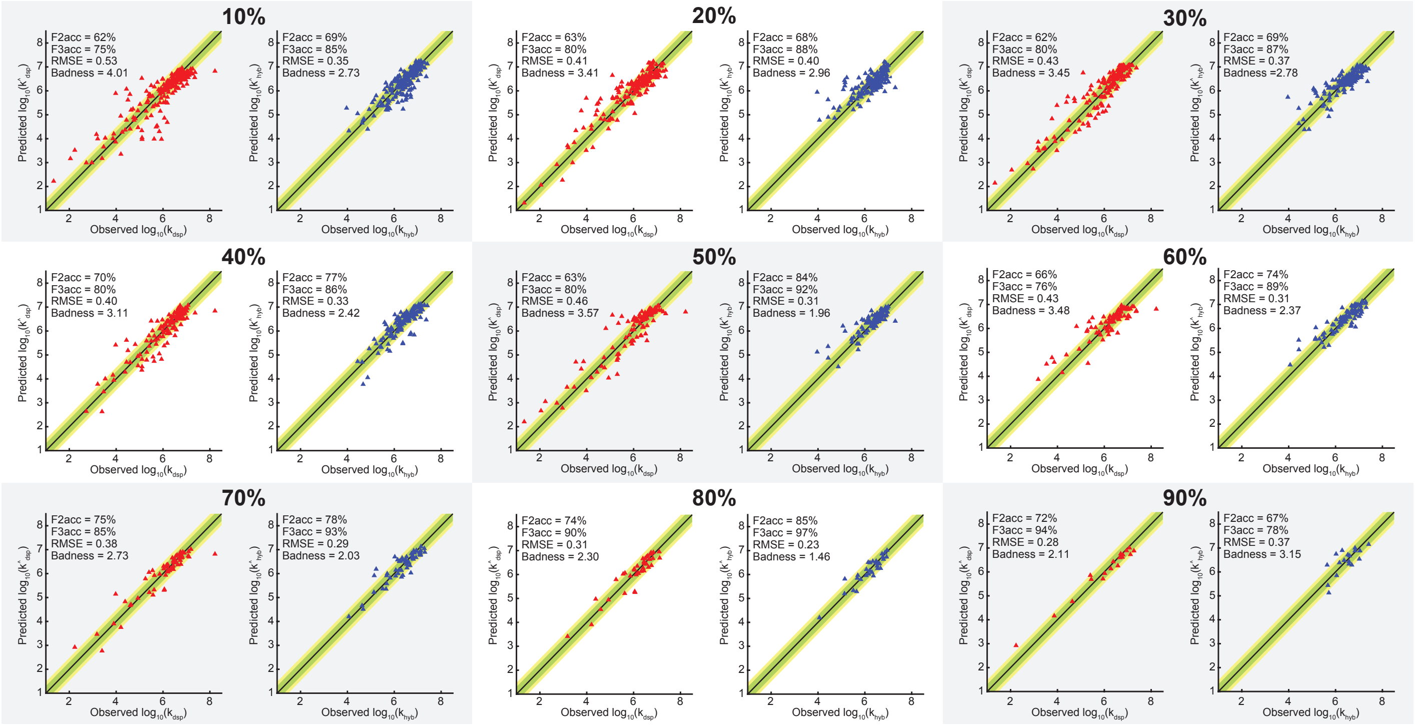

FIG. S37: DLM prediction accuracy using different size fractions of the data as the training set. Each pair of subfigures show the DLM predicted log rate constant vs. observed rate constant for strand displacement (left, red) and hybridization (right, blue). Green shading marks the zones where the predicted and the observed read depth agreed to within a factor of 2; yellow shading shows agreement to within factor of 3.

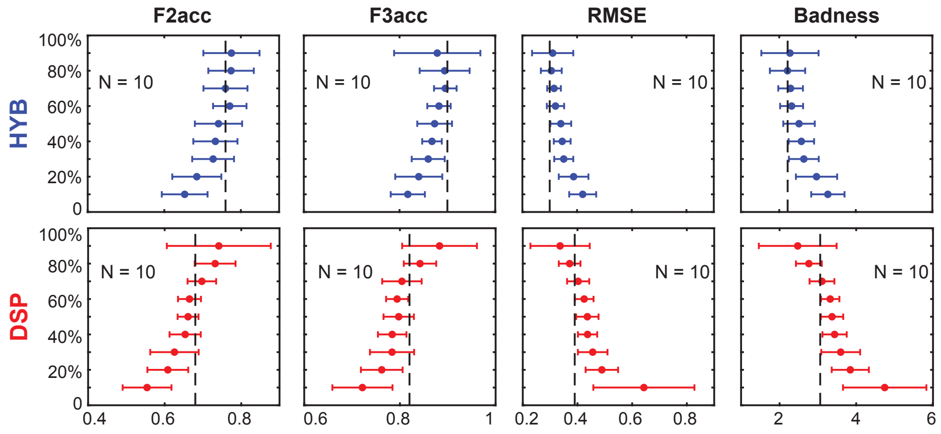

FIG. S38: Summary of DLM prediction accuracy using N=10 training sets for each training set size. Dots show the mean values, and the error bars show ±1 standard deviation.

DLM Generalization to Different Temperatures. To consider whether DLM accurately generalizes to different temperatures, we used our data for reactions at 37° and 55° as a training set, and characterized DLM predicted rate constants for 28°C and 46°C. As in the LOCO studies previously mentioned, we did not include in the training set the experiments using the same DNA sequences as the 28°C and 46°C experiments. Our results in Fig. S39 show that the DLM predicted rate constants have similar (or better) accuracy than our LOCO studies. Thus, the DLM appears to generalize quite well to different temperatures, including both interpolation (46°C) and extrapolation (28°C).

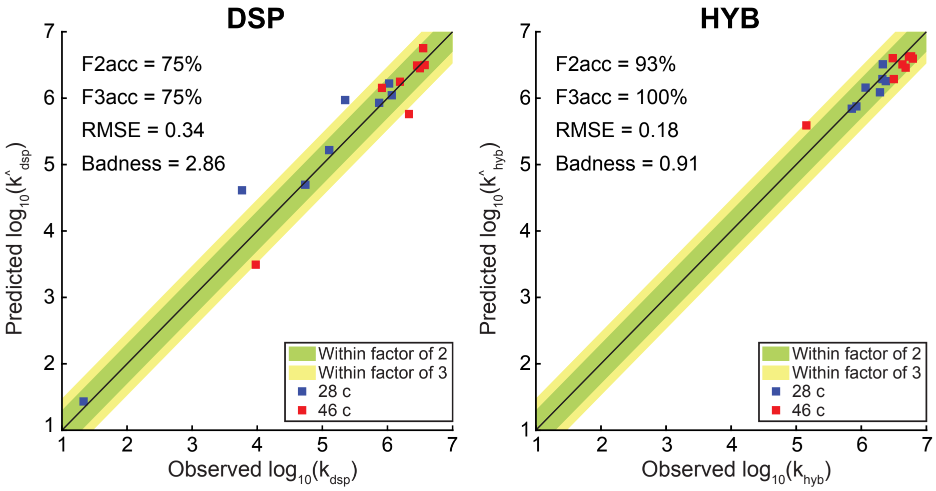

FIG. S39: DLM predictions of 28°C and 46°C rate constants based on training against 37°C and 55°C data. The training set does not include the experiments using the same DNA sequences as the 28°C and 46°C experiments. Green shading marks the zones where the predicted and the observed read depth agreed to within a factor of 2; yellow shading shows agreement to within factor of 3.

DLM Performance on Strand Displacement Reactions with Different Toehold Strengths. The length of the single-stranded toeholds that initiate the strand displacement reactions were designed to be all roughly -10 kcal/mol, because previous studies have shown that strand displacement kinetics saturate for this length toehold [5]. Based on our understanding of machine learning models, we do not expect that the DLM or WNV models would adequately predict the rate constants of strand displacement reactions with significantly shorter toehold lengths, given the lack of similar examples in the training data set.

Fig. S40AC shows the prediction performance of the DLM and WNV models, respectively, on the data from ref. [5], using our current dataset as the training set. Both models predict the rate constants very poorly, with little predicted rate constant dependence on the toehold length.

We next trained both the DLM and the WNV using our dataset, plus part of the dataset from ref. [5] (black dots). Fig. S40D shows the the WNV is able to quite accurately predict strand displacement rate constants for the remaining reactions. On the other hand, the DLM learns more slowly: Fig. S40B shows that the DLM now understands that shorter toeholds contribute to slower predicted reaction rate constants, but underestimates the dependence.

Our interpretation of this result is that the DLM has to contend with many observed data points in its training that it cannot predict perfectly, and thus considers that some labeled instances may include significant measurement error. The small number of DNA target sequences with short toeholds yielding very small rate constants thus has less impact on predictions than the large number of training examples with long toeholds and large rate constants. In contrast, the WNV model assigns more weight to these few new examples, because it considers them more relevant in our constructed feature space.

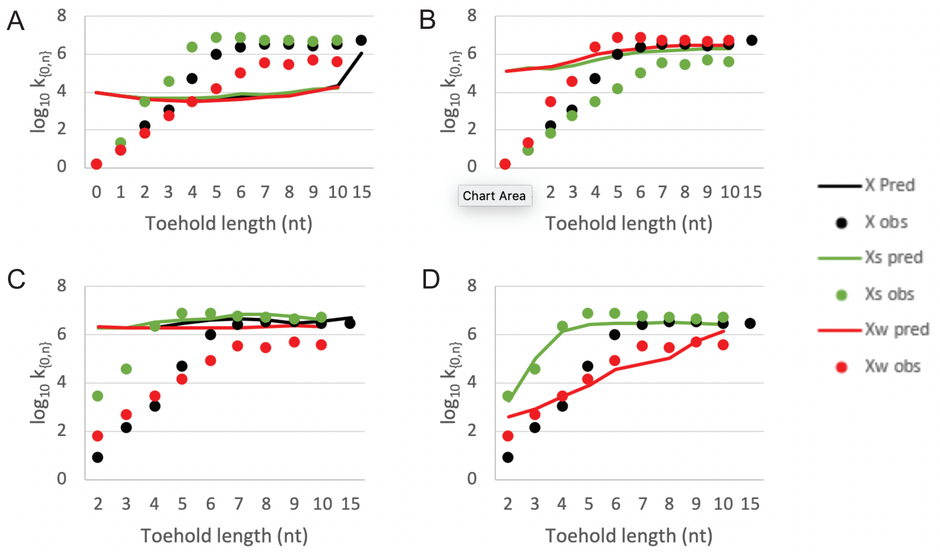

FIG. S40: Rate constant prediction for strand displacement reactions with shorter toeholds, based on data from ref. [5]. **(A)** DLM prediction using only the data from this manuscript as training data. **(B)** DLM prediction using the data from this manuscript plus the 4-letter alphabet toehold data (black dots) as training data. **(C)** WNV prediction using only the data from this manuscript as training data. **(D)** WNV prediction using the data from this manuscript plus the 4-letter alphabet toehold data (black dots) as training data.

Name	Complement	Target	$\log(k_{dsp})$
X(0:0)	CGTAGGGTATTGAATGAGGG	CCCTCATTCAATACCCTACG	0.146128
X(0:1)	ACGTAGGGTATTGAATGAGGG	CCCTCATTCAATACCCTACGT	0.912222
X(0:2)	GACGTAGGGTATTGAATGAGGG	CCCTCATTCAATACCCTACGTC	2.158362
X(0:3)	AGACGTAGGGTATTGAATGAGGG	CCCTCATTCAATACCCTACGTCT	3.033424
X(0:4)	GAGACGTAGGGTATTGAATGAGGG	CCCTCATTCAATACCCTACGTCTC	4.703291
X(0:5)	GGAGACGTAGGGTATTGAATGAGGG	CCCTCATTCAATACCCTACGTCTCC	5.984077
X(0:6)	TGGAGACGTAGGGTATTGAATGAGGG	CCCTCATTCAATACCCTACGTCTCCA	6.372912
X(0:7)	ATGGAGACGTAGGGTATTGAATGAGGG	CCCTCATTCAATACCCTACGTCTCCAT	6.507856
X(0:8)	CATGGAGACGTAGGGTATTGAATGAGGG	CCCTCATTCAATACCCTACGTCTCCATG	6.498311
X(0:9)	ACATGGAGACGTAGGGTATTGAATGAGGG	CCCTCATTCAATACCCTACGTCTCCATGT	6.44248
X(0:10)	GACATGGAGACGTAGGGTATTGAATGAGGG	CCCTCATTCAATACCCTACGTCTCCATGTC	6.451786
X(0:15)	GAAGTGACATGGAGACGTAGGGTATTGAATGAGGG	CCCTCATTCAATACCCTACGTCTCCATGTCACTTC	6.679428
Xs(0:1)	GCGTAGGGTATTGAATGAGGG	CCCTCATTCAATACCCTACGC	1.267172
Xs(0:2)	GGCGTAGGGTATTGAATGAGGG	CCCTCATTCAATACCCTACGCC	3.436163
Xs(0:3)	GGGCGTAGGGTATTGAATGAGGG	CCCTCATTCAATACCCTACGCCC	4.558709
Xs(0:4)	CGGGCGTAGGGTATTGAATGAGGG	CCCTCATTCAATACCCTACGCCCG	6.332438
Xs(0:5)	GCGGGCGTAGGGTATTGAATGAGGG	CCCTCATTCAATACCCTACGCCCGC	6.846337
Xs(0:6)	GGCGGGCGTAGGGTATTGAATGAGGG	CCCTCATTCAATACCCTACGCCCGCC	6.895423
Xs(0:7)	CGGCGGGCGTAGGGTATTGAATGAGGG	CCCTCATTCAATACCCTACGCCCGCCG	6.7348
Xs(0:8)	GCGGCGGGCGTAGGGTATTGAATGAGGG	CCCTCATTCAATACCCTACGCCCGCCGC	6.724276
Xs(0:9)	GGCGGCGGGCGTAGGGTATTGAATGAGGG	CCCTCATTCAATACCCTACGCCCGCCGCC	6.64836
Xs(0:10)	CGGCGGCGGGCGTAGGGTATTGAATGAGGG	CCCTCATTCAATACCCTACGCCCGCCGCCG	6.716838
Xw(0:1)	TCGTAGGGTATTGAATGAGGG	CCCTCATTCAATACCCTACGA	0.879096
Xw(0:2)	ATCGTAGGGTATTGAATGAGGG	CCCTCATTCAATACCCTACGAT	1.799341
Xw(0:3)	AATCGTAGGGTATTGAATGAGGG	CCCTCATTCAATACCCTACGATT	2.685742
Xw(0:4)	AAATCGTAGGGTATTGAATGAGGG	CCCTCATTCAATACCCTACGATTT	3.431364
Xw(0:5)	TAAATCGTAGGGTATTGAATGAGGG	CCCTCATTCAATACCCTACGATTTA	4.176091
Xw(0:6)	ATAAATCGTAGGGTATTGAATGAGGG	CCCTCATTCAATACCCTACGATTTAT	4.941511
Xw(0:7)	AATAAATCGTAGGGTATTGAATGAGGG	CCCTCATTCAATACCCTACGATTTATT	5.49276
Xw(0:8)	TAATAAATCGTAGGGTATTGAATGAGGG	CCCTCATTCAATACCCTACGATTTATTA	5.428135
Xw(0:9)	ATAATAAATCGTAGGGTATTGAATGAGGG	CCCTCATTCAATACCCTACGATTTATTAT	5.671173
Xw(0:10)	TATAATAAATCGTAGGGTATTGAATGAGGG	CCCTCATTCAATACCCTACGATTTATTATA	5.571709

TABLE I: Sequences and rate constants used in ref [5].

Supplementary Note 4. Contributions to the DLM Performance

Our DLM model used local features as input of RNN and results from RNNs were fed into the downstream FFNN together with 4 global features. We studied the importance of each feature by individually removing them from the default model, and then evaluating their impact on model performance. In the main text, we only summarize the comprehensive results of RMSEs, and here we present all the prediction accuracy figures of reduced models.

Local Feature Ablation.

Local features were features used in RNN input for calculating RNN node values. Removing one feature meant that we masked values of this feature at all nucleotides with 0, so that it should have no contribution to the RNN learning process. Fig. S41 is shown correspondingly to main text Fig. 5. Specifically, we used a binary code for representing base identity ($T = [0, 0]$, $C = [0, 1]$, $A = [1, 0]$, $G = [1, 1]$), and they were all masked to be 0 when sequence identity was removed.

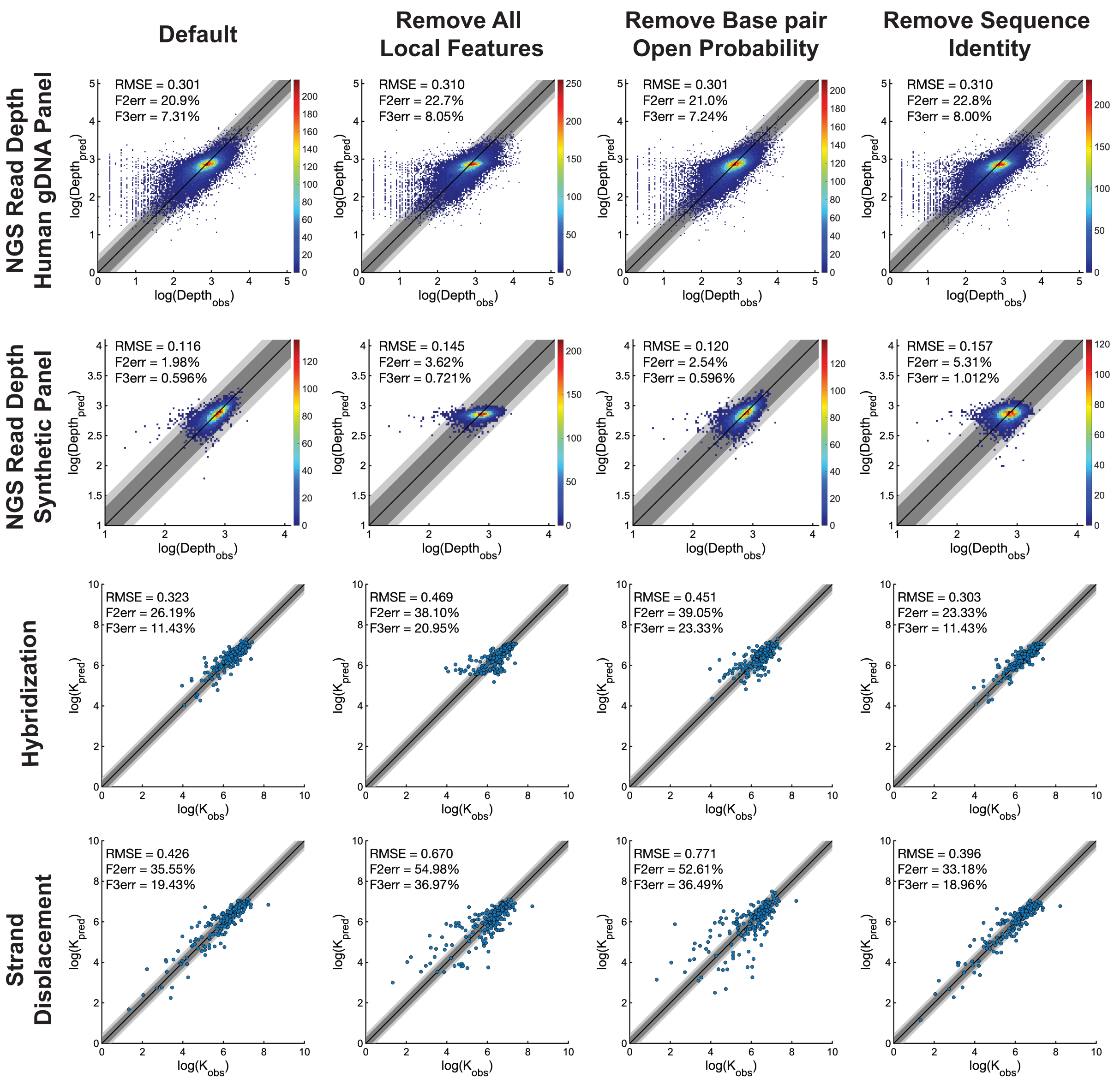

FIG. S41: **Ablation of local RNN features**. The first column shows the DLM 20-fold cross validation performance of the model with all features included for reference, as also presented in the main text. Second, third and forth columns show the effect of removing all the local features, removing the feature base pair open probability p_{unpaired} and the feature sequence identity respectively. Dark gray shading marks the zones where the predicted and the observed read depth agreed to within a factor of 2; light gray shows agreement to within factor of 3.

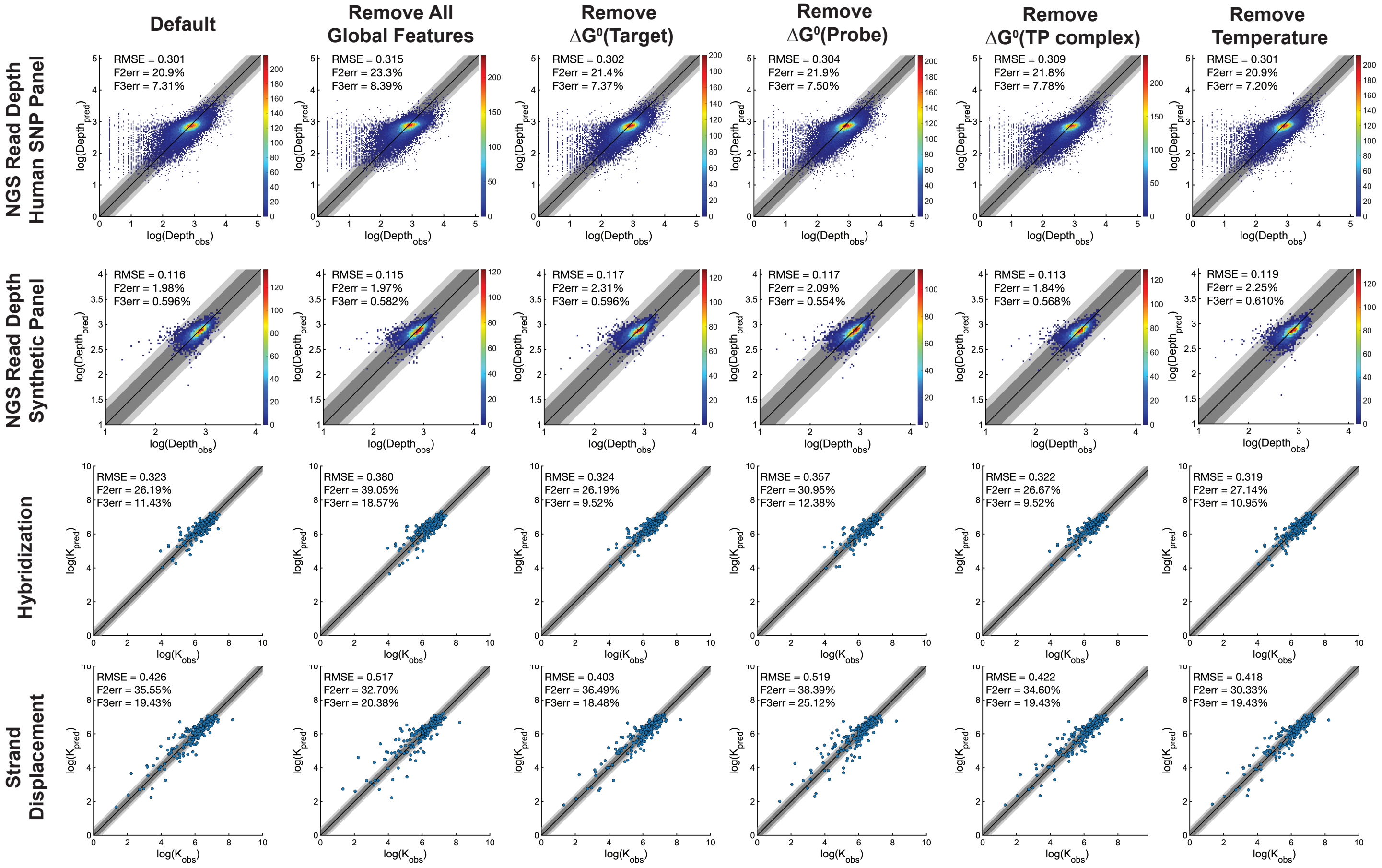

FIG. S42: **Ablation of global features**. The first column shows the DLM 20-fold cross validation performance of the model with all features included for reference, as also presented in the main text. The second column shows the effect of removing all 4 global features. Figures from the third to the end columns show the effect of removing each individual global feature: standard free energy of $\Delta G^{\circ}(T)$, standard free energy of capture probe$\Delta G^{\circ}(P)$, standard free energy of TP complex $\Delta G^{\circ}(TP)$, and temperature. Dark gray shading marks the zones where the predicted and the observed read depth agreed to within a factor of 2; light gray shows agreement to within factor of 3.

Global Feature Ablation.

Our global feature set was chosen based on the consideration that the model should be provided with information detailing the conditions and energy scales of the reaction. Removing temperature from global features did not change predictions on NGS read depth. This was expected considering that both NGS experiments were performed at the same temperature. Fig. S42 shows how removing all and individual global features affected overall performance.

Supplementary Note 5. Random Guess Models of Kinetics

As an alternative to the DLM and WNV models, in this section we illustrate the performance of two different naive, random-guess models. One possible approach is to estimate a distribution of rates from the data and make random predictions by sampling from this distribution. However, applying this approach results in significantly worse performance when compared to the DLM and WNV models we present, where only 35% of the HYB data and 37% of the DSP rates are predicted within a factor of three (Fig. S43AB). Another disadvantage of such an approach would be that a different random rate would be predicted when the model is applied repeatedly to the same sequence, making it not suitable in practice.

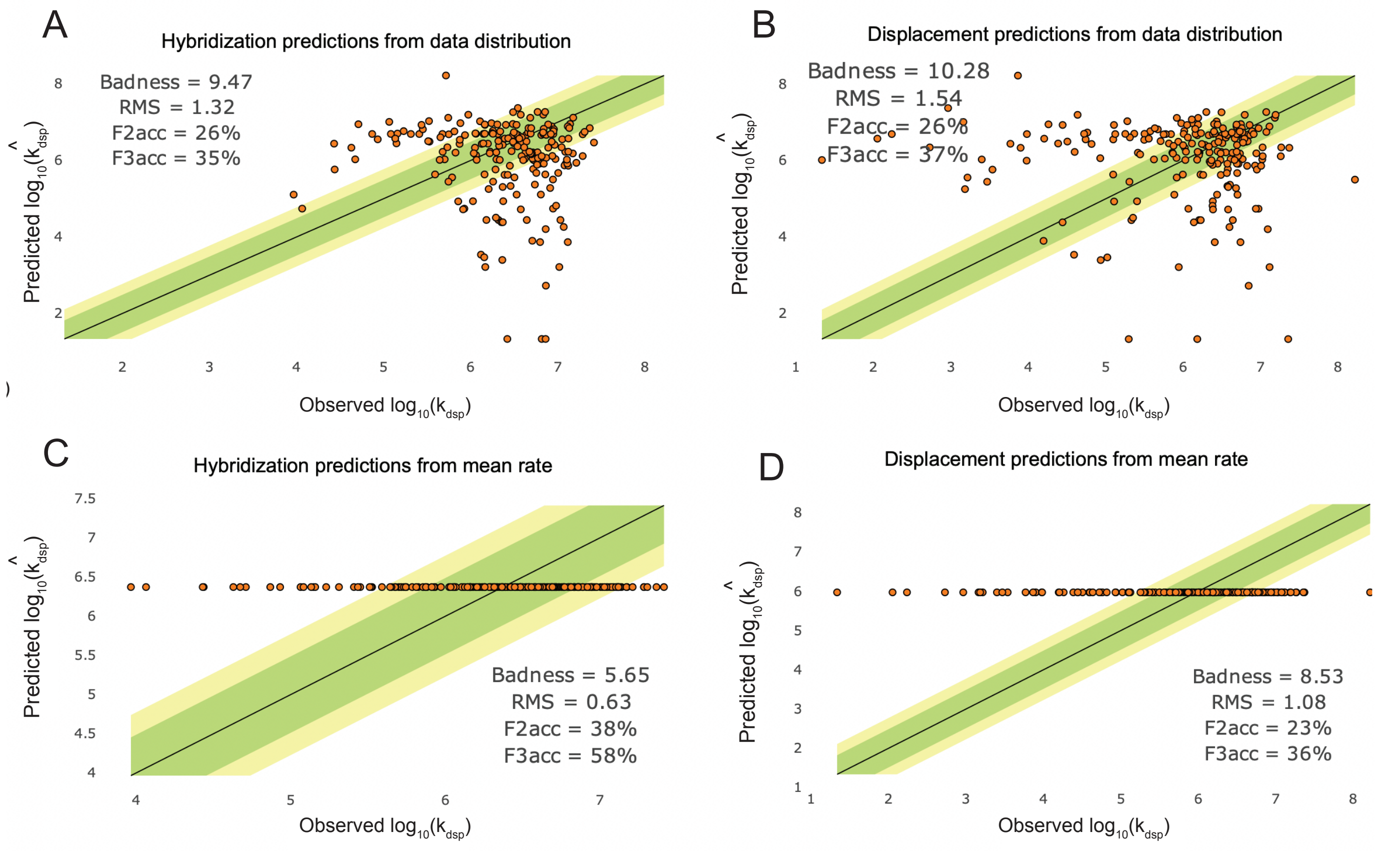

FIG. S43: Experimental strand displacement kinetic traces for the three new groups of DNA targets. Also shown are best-fit traces based on the different reaction models.

Another alternative could be to always predict the mean reaction rate as computed from the experimental data. This approach performs better than random sampling with 58% of the HYB data and 36% of the DSP rates are predicted within a factor of three (Fig. S43CD). However, this is still significantly worse than the DLM model reported in the paper.

These results indicate that naive models, either based on an empirical distribution or mean reaction rates, cannot achieve the performance of the DLM or WNV models. It is also worth noting that cross-validation was not applied when experimenting with these naive models. Withholding training data for testing is likely to further negatively impact the performance of the models when the empirical distribution or mean value does not include the value to be predicted

Sequence distance. We illustrate the distribution of DNA sequences used for the experiments reported in this paper by computing all pairwise Levenshtein distances - the number of single-character edits such as insertions, deletions or substitutions required to convert one sequence into another. The smallest distance between two distinct sequences is 4 and the largest is 30 with a mean distance of 21.839 (Fig. S44). These results indicate that the 36 nt long DNA molecules are not overly similar in terms of sequence, although they share certain properties in terms of secondary structures.

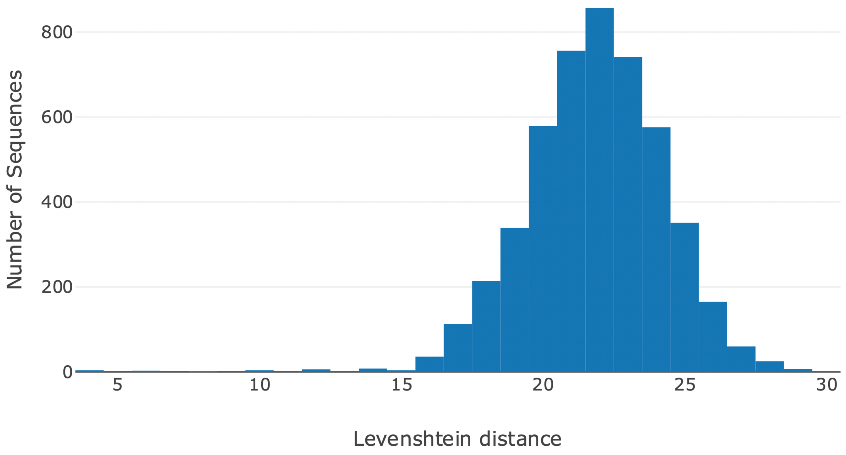

FIG. S44: Experimental strand displacement kinetic traces for the three new groups of DNA targets. Also shown are best-fit traces based on the different reaction models.

[1] R. M. Dirks, N. A. Pierce, A partition function algorithm for nucleic acid secondary structure including pseudoknots. Journal of computational chemistry, 24(13), 1664-1677 (2003).

[2] J. SantaLucia, D. Hicks, The Thermodynamics of DNA Structural Motifs. *Ann. Rev. Biochem.* **33,** 415-440 (2004).

[3] J. N. Zadeh, C. D. Steenberg, J. S. Bois, B. R. Wolfe, M. B. Pierce, A. R. Khan, N. A. Pierce, Nupack: analysis and design of nucleic acid systems. Journal of computational chemistry, 32(1), 170-173 (2011).

[4] K. Cho, B. Van Merrienboer, C. Gulcehre, D. Bahdanau, F. Bougares, H. Schwenk, Y. Bengio, Learning phrase representations using RNN encoder-decoder for statistical machine translation. arXiv preprint arXiv:1406.1078 (2014).

[5] D. Y. Zhang, E. Winfree, Control of DNA strand displacement kinetics using toehold exchange. Journal of the American Chemical Society, 131(47), 17303-17314 (2009).

[6] J. X. Zhang, J. Z. Fang, W. Duan, L. R. Wu, A. W. Zhang, N. Dalchau, B. Yordanov, R. Petersen, A. Phillips, D. Y. Zhang, Predicting DNA hybridization kinetics from sequence. Nature Chemistry, 10, 91-98 (2018).

[7] J. S. Wang, D. Y. Zhang, Simulation-guided DNA probe design for consistently ultraspecific hybridization. Nature chemistry, 7(7), 545 (2015).

[8] X. Glorot, Y. Bengio, Understanding the difficulty of training deep feedforward neural networks. In Proceedings of the thirteenth international conference on artificial intelligence and statistics, 249-256, (2010).

[9] B. Lakshminarayanan, A. Pritzel, C. Blundell, C. Simple and scalable predictive uncertainty estimation using deep ensembles. In Advances in Neural Information Processing Systems, 6402-6413, (2017).

[10] M. Pinedo, Scheduling: Theory, Algorithms and Systems, 2nd ed, Prentice Hall, Englewood Cliffs, NJ (2002).

[11] N.R. Smith Draper, Applied regression analysis. (2nd Ed.) New York: Wiley (1981).
