# Supplementary material for: A deep learning model for predicting next-generation sequencing depth from DNA sequence
Source: Nat Commun. 2021 Jul 19;12:4387. doi: 10.1038/s41467-021-24497-8 (PMC8290051; doi:10.1038/s41467-021-24497-8)
Supplement: Supplementary file 3 — Description of Additional Supplementary Files [file 41467_2021_24497_MOESM3_ESM.docx]

**Description of Additional Supplementary Files**

File name: Supplementary Data 1

Description: Probes of SNP panel

File name: Supplementary Data 2

Description: Probes of lncRNA panel

File name: Supplementary Data 3

Description: Probes of synthetic panel

File name: Supplementary Data 4

Description: Sequences of strand displacement experiments

File name: Supplementary Data 5

Description: Sequences of hybridization experiments

File name: Supplementary Data 6

Description: Features of WNV model
